# Dynamic regulation of P-TEFb by 7SK snRNP is integral to the DNA damage response to regulate chemotherapy sensitivity

**DOI:** 10.1016/j.isci.2022.104844

**Published:** 2022-08-04

**Authors:** Yin Fang, Yan Wang, Benjamin M. Spector, Xue Xiao, Chao Yang, Ping Li, Yuan Yuan, Ping Ding, Zhi-Xiong Xiao, Peixuan Zhang, Tong Qiu, Xiaofeng Zhu, David H. Price, Qintong Li

**Affiliations:** 1 Departments of Pediatrics and Obstetrics & Gynecology, West China Second University Hospital, Key Laboratory of Birth Defects and Related Diseases of Women and Children, Ministry of Education, Development and Related Diseases of Women and Children Key Laboratory of Sichuan Province, Center of Growth, Metabolism and Aging, College of Life Sciences, State Key Laboratory of Biotherapy and Collaborative Innovation Center of Biotherapy, Sichuan University, Chengdu 610041, China; 2 Department of Biochemistry, University of Iowa, Iowa City, IA 52242, USA; 3 Division of Bioinformatics, Sichuan Cunde Therapeutics, Chengdu 610093, China; 4 Non-coding RNA and Drug Discovery Key Laboratory of Sichuan Province, Chengdu Medical College, Chengdu 610500, China; 5 Center of Growth, Metabolism and Aging, Key Laboratory of Bio-Resource and Eco-Environment of Ministry of Education, College of Life Sciences, Sichuan University, Chengdu 610064, China

**Keywords:** Molecular biology, Molecular mechanism of gene regulation, Cancer

## Abstract

Testicular germ cell tumors and closely related embryonal stem cells are exquisitely sensitive to cisplatin, a feature thought to be linked to their pluripotent state and p53 status. It remains unclear whether and how cellular state is coordinated with p53 to confer cisplatin sensitivity. Here, we report that positive transcription elongation factor b (P-TEFb) determines cell fate upon DNA damage. We find that cisplatin rapidly activates P-TEFb by releasing it from inhibitory 7SK small nuclear ribonucleoprotein complex. P-TEFb directly phosphorylates pluripotency factor estrogen-related receptor beta (ESRRB), and induces its proteasomal degradation to enhance pro-survival glycolysis. On the other hand, P-TEFb is required for the transcription of a substantial portion of p53 target genes, triggering cell death during prolonged cisplatin treatment. These results reveal previously underappreciated roles of P-TEFb to coordinate the DNA damage response. We discuss the implications for using P-TEFb inhibitors to treat cancer and ameliorate cisplatin-induced ototoxicity.

## Highlights

• P-TEFb regulates pro-survival and pro-death pathways during DNA damage response
• P-TEFb promotes ESRRB proteasomal degradation to enhance pro-survival glycolysis
• P-TEFb induces a substantial portion of p53 target genes to trigger cell death
• Chemical inhibitors of P-TEFb blocks cisplatin- or UV-induced cell death

## Graphical abstract

**Figure:**
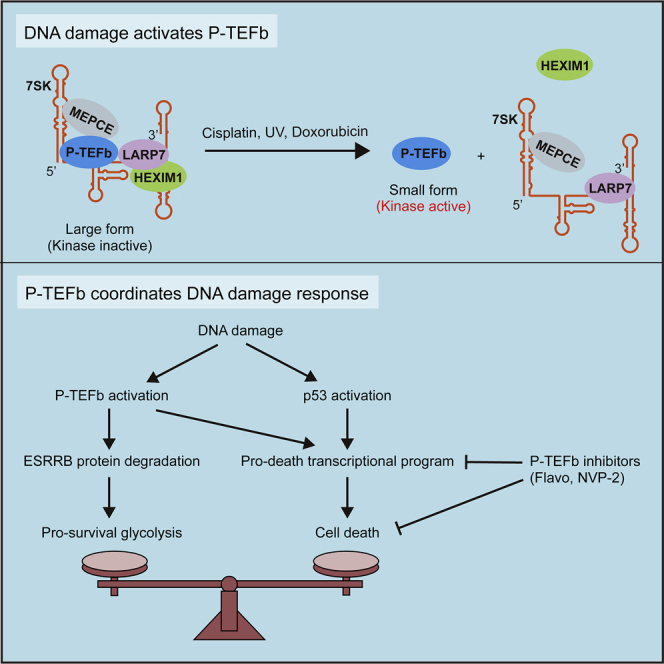

## Introduction

Testicular germ cell tumor (TGCT) is the most common malignancy among young men with increasing incidence over the past four decades (Bray et al., 2006; Ruf et al., 2014; Trabert et al., 2015). A high cure rate is achieved in TGCTs due to their exceptional sensitivity to cisplatin (Oldenburg et al., 2013). Nevertheless, ∼10% patients exhibit cisplatin resistance and their 5-year survival is 10%–15% (Einhorn et al., 2007; Oechsle et al., 2011). In addition, cisplatin-induced hearing loss, or ototoxicity, is prevalent among TGCT survivors due to tumor incidence at a young age and a high cure rate, resulting in a multifaceted decrease in quality of life (Frisina et al., 2016; Knight et al., 2005). More than 60% of patients experience progressive cisplatin-induced hearing loss, presenting a serious clinical challenge for TGCT survivors (Freyer et al., 2020). The molecular mechanism of cisplatin-induced ototoxicity is poorly understood (Freyer et al., 2019; Gentilin et al., 2019), but can be explained by the fact that cisplatin accumulates in the stria vascularis region of the cochlea in both mice and humans (Breglio et al., 2017). This highly specialized tissue produces the endolymph, a unique fluid that bathes the sensory hair cells of the inner ear and is crucial for auditory transduction (Korver et al., 2017).

The exquisite sensitivity or resistance of TGCTs to cisplatin is not completely understood, but thought to be associated with their embryonal stem cell-like cellular states (Oosterhuis and Looijenga, 2019). Pathological assessments and molecular profiling studies collectively support the notion that the seminoma TGCT subtype arises from pluripotent primordial germ cells/gonocytes, and the nonseminoma subtype from embryonic stem cells (Oosterhuis and Looijenga, 2005; Sperger et al., 2003). Similar to their malignant counterparts, these pluripotent embryonal stem cells exhibit hypersensitivity to DNA damage, a characteristic thought to limit the risk of passing mutations to the next generation (Liu et al., 2013). Notably, TGCTs largely retain the molecular and cellular features of their embryonal precursors, such as the expression of pluripotency transcription factors *POU5F1* (commonly known as *OCT4*) and *Nanog* (Oosterhuis and Looijenga, 2019). Differentiation either by *OCT4* knockdown or retinoic acid treatment induces resistance to cisplatin in both TGCTs and embryonal stem cells (Gutekunst et al., 2013). This phenomenon closely resembles clinical observations in that undifferentiated TGCT subtypes (high *OCT4* and *Nanog* expression) are highly sensitive, whereas differentiated teratomas (negative for *OCT4* and *Nanog* expression) are fully resistant to cisplatin treatment (Litchfield et al., 2016). In addition, the heightened acute DNA damage response in TGCT and embryonal stem cells are thought to be determined by their wild-type *p53* status to induce mitochondria-mediated cell death (Cavallo et al., 2013). Indeed, mutations in *p53* and *MDM2* were observed in 21.3% of men with cisplatin-resistant TGCTs versus 3% in those with cisplatin-sensitive TCGTs (Bagrodia et al., 2016). This result also means that the mechanisms remain undefined in ∼80% of cisplatin-resistant TGCTs, but are likely independent of p53 status. Moreover, p53 function is not required to maintain undifferentiated state of embryonal stem cells or TCGTs (Li and Belmonte, 2017). Thus, the molecular mechanisms linking undifferentiated cellular state to cisplatin hypersensitivity or resistance remain unclear.

Positive transcription elongation factor b (P-TEFb), composed of CDK9 catalytic subunit and cyclin T regulatory subunit, is a well-established regulator of RNA polymerase II (Zhou et al., 2012). The best-characterized substrate of P-TEFb is the C-terminal domain of the largest subunit of RNA polymerase II. Because P-TEFb is required for the transcription of most protein-coding genes, its kinase activity is tightly regulated under homeostatic conditions (Guo and Price, 2013). Most cellular P-TEFb is sequestered and inactivated by 7SK small nuclear ribonucleoprotein complex (7SK snRNP) (He et al., 2008; Krueger et al., 2008; Li et al., 2005; Markert et al., 2008; Michels et al., 2003, 2004; Nguyen et al., 2001; Yang et al., 2001; Yik et al., 2003). It is generally believed that increased P-TEFb activity globally enhances RNA polymerase II transcriptional output, thus should promote cellular growth and proliferation. Under this assumption, there are growing interests in developing specific P-TEFb pharmaceutical inhibitors as anticancer reagents (Wu et al., 2020). However, it is worth noting that loss of 7SK snRNP in humans is associated with developmental delay rather than overgrowth (Alazami et al., 2012). In addition, it has been known for two decades that acute DNA damage signals such as ultraviolet radiation rapidly release P-TEFb from 7SK snRNP, and therefore increase cellular P-TEFb kinase activity (Nguyen et al., 2001). This phenomenon is paradoxical because P-TEFb kinase activity promotes gene transcription, but global transcription is downregulated during the DNA damage response (Lans et al., 2019). Furthermore, the transcription of p53 target genes, a major DNA damage-induced cellular response, is thought to be independent of P-TEFb activity (Gomes et al., 2006). Thus, the significance of dynamic regulation of P-TEFb by 7SK snRNP under acute DNA damage conditions awaits elucidation.

We postulated that differentiation status-associated cisplatin sensitivity/resistance and cisplatin-induced ototoxicity might be linked by shared molecular targets. In this regard, we leveraged data from human genetics studies (Korver et al., 2017). Among ∼250 human genes associated with the hereditary hearing loss condition (https://hereditaryhearingloss.org), estrogen-related receptor beta (*ESRRB*) stands out for several reasons. The expression of *ESRRB* in the inner ear is localized in in the stria vascularis region of the cochlea (Chen and Nathans, 2007; Collin et al., 2008), the exact anatomic site where cisplatin accumulates in mice and humans (Breglio et al., 2017). Loss-of-function mutations of *ESRRB* are causatively linked to hereditary hearing loss in humans (Collin et al., 2008; Weber et al., 2014). Consistently, conditional knockout of *ESRRB* in the stria vascularis region leads to hearing loss in mice (Chen and Nathans, 2007). On the other hand, ESRRB is known to promote the maintenance of naive pluripotency in embryonic stem cells and primordial germ cells (Adachi et al., 2018; Festuccia et al., 2016; Okamura et al., 2019; Zhang et al., 2018), precursors to TGCTs (Oosterhuis and Looijenga, 2019). Loss of ESRRB expression switches embryonic stem cells from naive to the primed state without altering the expression of OCT4 or SOX2 (Festuccia et al., 2018). How ESRRB activity is regulated by intrinsic and extracellular signaling pathways is not well understood. In this study, we started by investigating whether cisplatin treatment regulates ESRRB, and found that P-TEFb coordinates ESRRB and p53 activities to determine cell fate upon acute DNA damage.

## Results

Seminoma and nonseminoma are two major malignant TGCT subtypes in humans. Of note, there is only one potential seminoma TGCT cell line available (de Jong et al., 2008), and currently there are no suitable *in vivo* mouse genetic models to recapitulate undifferentiated cellular features of human TGCTs (Oosterhuis and Looijenga, 2005, 2019). Thus, we decided to use embryonic stem cells (ESCs) as a model system in present study, because the exquisite cisplatin sensitivity of TGCTs is thought to be inherited from their normal embryonal counterparts (Oosterhuis and Looijenga, 2019). In addition, the cellular states of ESCs can be manipulated to reflect pluripotent and differentiated TGCT subtypes (Oosterhuis and Looijenga, 2005, 2019). ESCs can be converted into primordial germ cells (Hayashi et al., 2008, 2011), the normal counterparts of seminomous TGCTs. On the other hand, ESCs can generate a mixture of embryonal carcinoma cells and teratomas in syngeneic mice (Evans, 2011), mimicking nonseminomatous TGCTs (Oosterhuis and Looijenga, 2019). More importantly, ESC and its derivatives share the same genetic background, thus provide a more defined system than genetically diverse TGCT cell lines.

### Acute DNA damage promotes the proteasomal degradation of Esrrb

**Figure 1:**
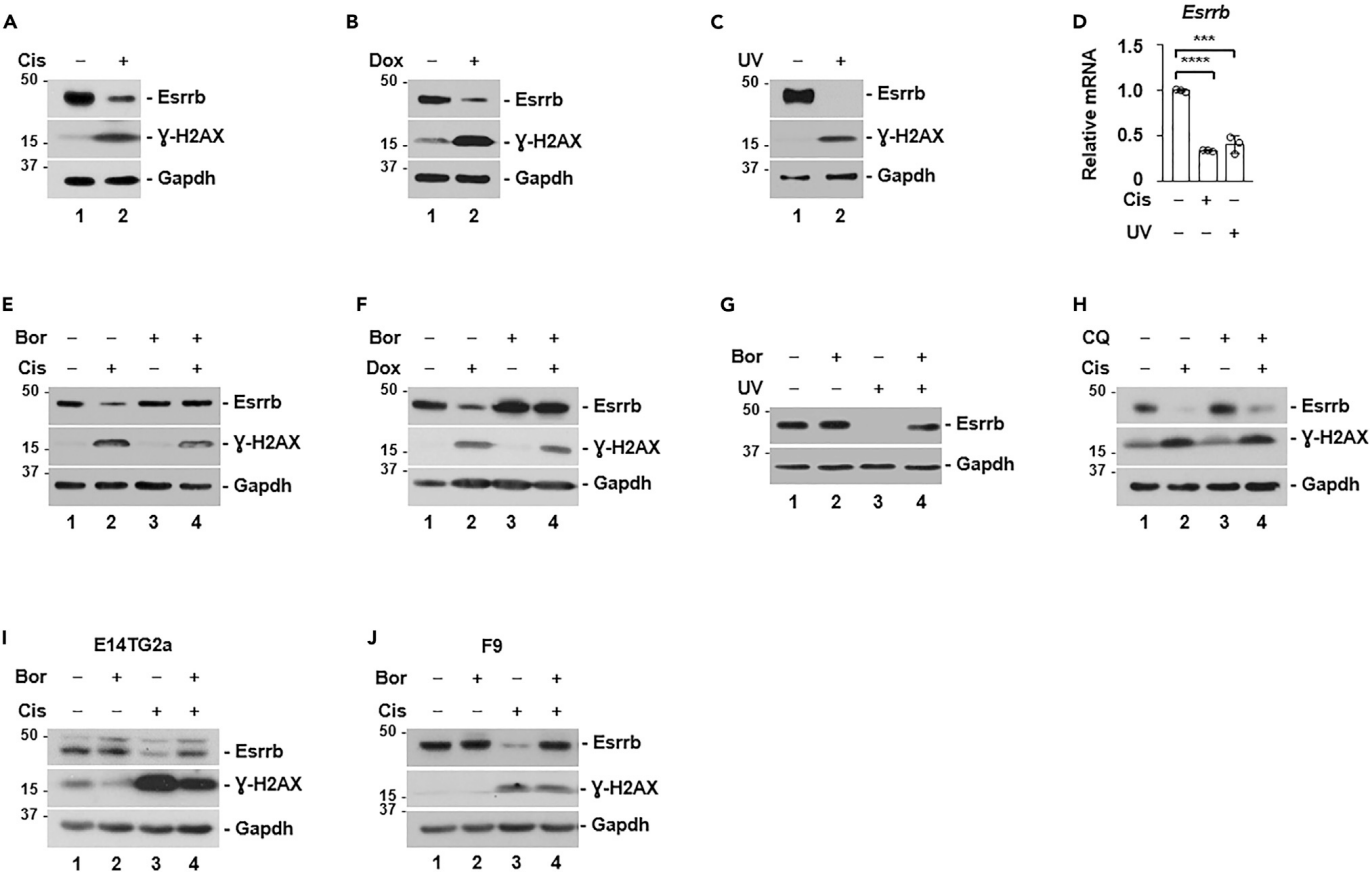
Acute DNA damage promotes the proteasomal degradation of Esrrb (A, B and C) Western blot analyses of indicated proteins from total cell lysate of R1/E ES cells, treated with or without cisplatin (Cis, 5 μM), doxorubicin (Dox, 1 μM), or ultraviolet radiation (UV, 10 J/m^2^). γ-H2AX is a marker of acute DNA damage. (D) qPCR analysis of Esrrb mRNA with or without cisplatin or UV (10 J/m^2^) treatment in R1/E ES cells (n = 3). The bar plot represents mean ± SD. ∗∗∗, p < 0.001; ∗∗∗∗, p < 0.0001 (unpaired *t* test). (E, F and G) Western blot analyses of indicated proteins from total cell lysate of R1/E ES cells, treated with cisplatin (Cis, 5 μM) (E), doxorubicin (Dox, 1 μM) (F), or UV (UV,10 J/m^2^) (G), in the absence or presence of the proteasomal inhibitor, bortezomib (Bor, 100 nM). (H) Western blot analyses of indicated proteins from total cell lysate of R1/E ES cells, treated with cisplatin in the absence or presence of the autophagy inhibitor, chloroquine (CQ, 25 μM). (I and J) Western blot analyses of indicated proteins from total cell lysate of E14TG2a ES cells (I) or F9 embryonal carcinoma cells (J), treated with or without cisplatin (Cis, 5 μM), in the absence or presence of the proteasomal inhibitor, bortezomib (Bor, 100 nM).

We investigated whether the expression of *Esrrb* could be regulated by acute DNA damage. For following experiments, we mostly used R1/E ES cell line, and key observations were also validated using E14TG2a (an ES cell line derived from a different genetic background than R1/E) and F9 embryonal carcinoma cell (the neoplastic stem cells of nonseminomas). The average maximal plasma concentration of cisplatin in patients after a single administration is 14.3 μM (Liston and Davis, 2017). In clinical anticancer regimens, cisplatin is administered for several rounds. Thus, the concentration in the stria vascularis of the cochlea is likely much higher, because this region accumulates cisplatin indefinitely both in mice and humans (Breglio et al., 2017). We chose 5 μM cisplatin for the following experiments because 5, 10, or 20 μM all reduced Esrrb protein expression in a pilot study. Esrrb protein level in R1/E cells was markedly reduced by cisplatin treatment (Figure 1A). The average maximal plasma concentration of doxorubicin in patients is 6 μM (Liston and Davis, 2017). We found that treatment with either doxorubicin (1 μM) or ultraviolet irradiation (UV, 10 J/m^2^) similarly reduced Esrrb protein expression (Figures 1B and 1C). As expected, the phosphorylation of the histone variant H2AX (γ-H2AX) was massively induced by cisplatin, doxorubicin, or UV, indicative of acute DNA damage response (Kinner et al., 2008). Because cisplatin, UV, and doxorubicin cause different types of DNA damage, these results indicated that Esrrb is regulated by cellular signaling pathways downstream of acute DNA damage. Quantitative polymerase chain reaction (qPCR) analyses revealed a moderate reduction in *Esrrb* mRNA level under these DNA damage conditions (Figure 1D). However, co-treatment with proteasomal inhibitor bortezomib (Figures 1E, 1F and 1G), but not autophagy inhibitor chloroquine (Figure 1H), largely blocked cisplatin, doxorubicin, or UV-induced reduction in Esrrb protein levels. Additionally, in E14TG2a and F9 cells, we found that cisplatin similarly induced the proteasomal degradation of Esrrb (Figures 1I and 1J). Thus, diverse acute DNA damage conditions induce the proteasomal degradation of Esrrb protein.

### Proteasomal degradation of Esrrb protein is not regulated by canonical DNA damage response pathways

In canonical DNA damage response, the apical kinases ATM (ataxia-telangiectasia mutated kinase), DNA-PK (DNA-dependent protein kinase), and ATR (ATM and Rad3-related) are activated to phosphorylates downstream effector kinases CHK1 and CHK2. These kinase cascades promote either cell survival or death dependent on the strength and extent of acute DNA damage (Blackford and Jackson, 2017).

We examined whether canonical DNA damage response was responsible for the proteasomal degradation of Esrrb induced by cisplatin. Pretreatment or co-treatment with chemical inhibitors of ATM (KU-60019), ATR (VE-821), and DNA-PK (NU7441) individually failed to prevent cisplatin-induced Esrrb degradation (Figure S1A). In fact, some of those inhibitors further promoted the degradation of Esrrb (Figures S1A and S1B). This is likely because they enhanced the degree of acute DNA damage, indicated by increased γ-H2AX. Pretreatment with dual or triple combination of these inhibitors also failed to suppress cisplatin-induced Esrrb degradation (Figure S1B). Likewise, chemical inhibition of CHEK1/2 (AZD7762) did not prevent Esrrb degradation (Figure S1C). Recent studies showed that p38 MAPK collaborates with ATM/ATR to regulate DNA damage response (Borisova et al., 2018). We found that treatment with p38 MAPK inhibitor (SB223580) did not prevent cisplatin-induced Esrrb degradation (Figure S1D). Finally, knockdown of Chk1 and Chk2 individually or in combination with their chemical inhibitor failed to block Esrrb degradation (Figures S1E, S1F, and S1G). We concluded that DNA damage-induced proteasomal degradation of Esrrb is independent of kinase cascades in canonical DNA damage response.

**Figure 2:**
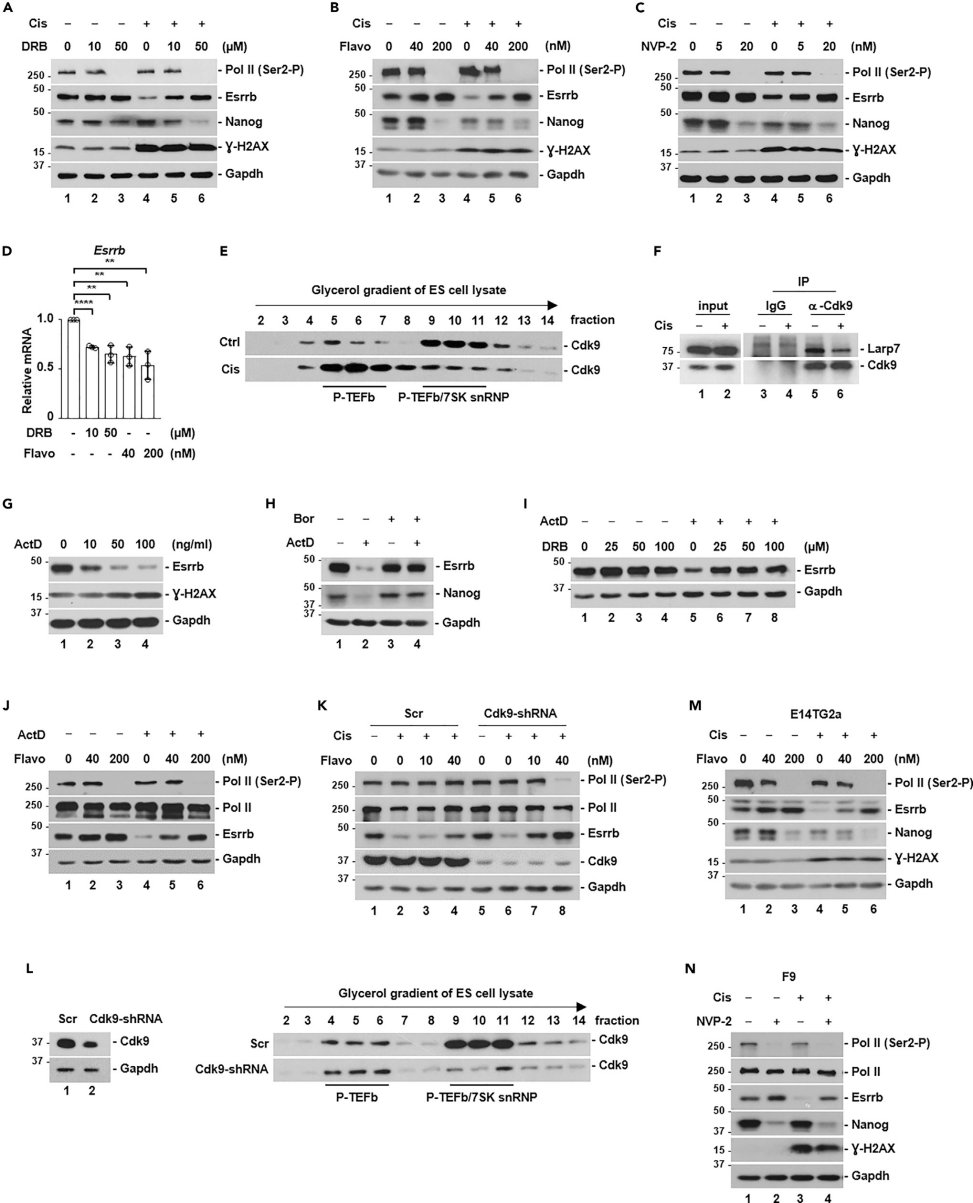
Cisplatin activates P-TEFb to promote Esrrb proteasomal degradation (A, B and C) Western blot analyses of indicated proteins from total cell lysate of R1/E ES cells, treated with or without cisplatin (Cis, 5 μM), in the presence or absence of chemical inhibitors of P-TEFb, DRB (A), flavopiridol (Flavo) (B) and NVP-2 (C). The Ser2 phosphorylation of RNA polymerase II CTD, Pol II (Ser2-P), is a well-established substrate of P-TEFb. (D) qPCR analysis of Esrrb mRNA with or without chemical inhibitors of P-TEFb, DRB, and flavopiridol. The bar plot represents mean ± SD. ∗∗, p < 0.01; ∗∗∗∗, p < 0.0001 (unpaired *t* test). (E) Linear glycerol gradient analysis of free P-TEFb and P-TEFb complexed with 7SK snRNP (P-TEFb/7SK snRNP), with or without cisplatin (Cis, 5 μM) treatment. DMSO was used a vehicle treatment control. (F) Analysis of the interaction between endogenous Cdk9 and Larp7 proteins treated with or without cisplatin (Cis, 5 μM) by anti-Cdk9 immunoprecipitation. (G) Western blot analyses of indicated proteins from total cell lysate of R1/E ES cells, treated with actinomycin D (ActD) of indicated concentration. ActD can activate P-TEFb by releasing it from 7SK snRNP. (H) Western blot analyses of indicated proteins from total cell lysate, treated with or without actinomycin D (ActD, 100 ng/mL), in the presence or absence of proteasomal inhibitor, bortezomib (Bor). (I and J) Western blot analyses of indicated proteins from total cell lysate, treated with or without actinomycin D (ActD, 100 ng/mL), in the presence or absence of P-TEFb chemical inhibitors, DRB (I) or flavopiridol (J). (K) The effect of Cdk9 knockdown by small-hairpin RNA (shRNA) on cisplatin-induced Esrrb proteasomal degradation. Scr, scrambled shRNA. (L) Linear glycerol gradient analysis of free P-TEFb and P-TEFb complexed with 7SK snRNP (P-TEFb/7SK snRNP) with or without Cdk9 knockdown (Cdk9-shRNA). Scr, scrambled shRNA. (M and N) The effect of P-TEFb chemical inhibitors on cisplatin-induced Esrrb degradation in E14TG2a ES cells (M) and F9 embryonal carcinoma cells (N), cisplatin (Cis, 5 μM).

The expression of Esrrb may be transcriptionally regulated by Nanog (Festuccia et al., 2012). We noticed that cisplatin, UV, or doxorubicin treatment reduced Nanog protein level to various degrees (Figures S2A, S2B, and S2C). A previous study suggested that acute DNA damage activates p53 to transcriptionally repress *Nanog* expression (Lin et al., 2005). These results indicated that the p53-Nanog axis might, at the level of transcription, contribute to cisplatin-induced reduction in Esrrb. However, we did not find evidence to support the notion that p53 represses Nanog protein expression under acute DNA damage conditions. Similar to the previous study (Lin et al., 2005), we found a 2-fold reduction in *Nanog* mRNA level after cisplatin or UV treatment in a p53-depedent manner (Figure S2D). However, proteasomal inhibitors fully restored Nanog protein level (Figures S2A, S2B, and S2C), indicating that protein stability rather than transcription is the main regulatory mechanism of Nanog expression under acute DNA damage conditions. Furthermore, both Nanog and Esrrb protein levels were decreased to a similar degree in p53 wild-type or knockout cell lines (Figure S2E), demonstrating that both proteins are not regulated by p53 under acute DNA damage conditions. p53 wild-type and knockout cell lines were highly similar in terms of expression levels of pluripotency factors, positive alkaline phosphatase staining, and overall transcriptome (Zhang et al., submitted), indicating that the reduction in Esrrb and Nanog protein levels was not caused by cellular differentiation. Activation of p53 is a major event downstream of ATM/ATR activation in cellular response to DNA damage. Thus, p53-independent regulation of Esrrb was also consistent with results showing that kinase cascades of canonical DNA damage response were not responsible for cisplatin-induced Esrrb protein degradation (Figure S1). Results presented below with CDK9 inhibitors also ruled out the possibility that cisplatin-induced Esrrb degradation was somehow caused by decreased Nanog protein level, because these inhibitors prevented Esrrb degradation but further reduced Nanog expression (Figures 2A, 2B, 2C, 2M, and 2N). Thus, under acute DNA damage conditions, Esrrb expression is not controlled by Nanog or canonical DNA damage response pathways hinged upon the ATM/ATR-p53 axis.

### Cisplatin activates P-TEFb to promote Esrrb proteasomal degradation

To elucidate the signaling pathways that regulate cisplatin-induced Esrrb proteasomal degradation, we carried out a screen with a panel of small-molecule inhibitors against members of CDK, MAPK, GSK, PKC, and PI3K families of protein kinases. We found that DRB (5,6-dichloro-1-β-D-ribofuranosyl-1H-benzimidazole), a widely used P-TEFb inhibitor (Bensaude, 2011), largely blocked cisplatin-induced Esrrb protein degradation (Figure 2A). Of note, Esrrb protein level was even restored by 10 μM of DRB (lane 5, Figure 2A), whereas the phosphorylation of well-established P-TEFb substrate, RNA polymerase II CTD, was minimally affected (lane 5, Figure 2A). Similarly, treatment with flavopiridol, a more potent and selective P-TEFb inhibitor (Bensaude, 2011), prevented cisplatin-induced Esrrb degradation (Figure 2B). NVP-2 is a recently developed P-TEFb inhibitor with high specificity against CDK9 among human kinome. The only other potential target of NVP-2 is CDK10, which is not a target of DRB or flavopiridol (Olson et al., 2018). Again, treatment with NVP-2 blocked cisplatin-induced Esrrb degradation (Figure 2C). The efficacy of these P-TEFb inhibitors was demonstrated by marked reduction in RNA Pol II CTD Ser-2 phosphorylation, a well-established P-TEFb substrate (Figures 2A, 2B, and 2C). Notably, none of these P-TEFb inhibitors prevented cisplatin-induced reduction in Nanog protein (Figures 2A, 2B, and 2C). In fact, these P-TEFb inhibitors alone reduced Nanog protein expression, likely due to much decreased Nanog mRNA expression (Figure S2F). In addition, all these P-TEFb inhibitors moderately reduced mRNA level of Esrrb (Figure 2D). Thus, cisplatin-induced Esrrb reduction was not caused by Nanog-mediated transcriptional control of Esrrb. We also used chemical inhibitors targeting other members of CDK protein family, including CDK1/2 (RO3306), CDK4/6 (Palbociclib), CDK7 (THZ1), and CDK12/13 (THZ531). The efficacy of these inhibitors was ascertained by cyclin B1 (encoded by Ccnb1) protein accumulation by CDK1/2 inhibition, elimination of phosphorylation in RB protein by CDK4/6 inhibition, reduction of Ser5 and Ser2 phosphorylation of RNA Pol II CTD by CDK7 and CDK12/13 inhibition, respectively. None of these inhibitors could block cisplatin-induced Esrrb degradation (Figure S3). These results indicated that cisplatin treatment activates P-TEFb kinase activity to promote Esrrb degradation.

Most cellular P-TEFb is sequestered and inactivated by 7SK snRNP. It is known that UV irradiation rapidly dissociates P-TEFb from 7SK snRNP, leading to increased P-TEFb kinase activity (Nguyen et al., 2001). Similarly, we found that cisplatin treatment released P-TEFb from 7SK snRNP (Figure 2E), and reduced the interaction between P-TEFb and Larp7 protein, the core constituent of 7SK snRNP (Figure 2F). Actinomycin D, an inhibitor of RNA Pol II transcription, is also known to activate P-TEFb by releasing it from 7SK snRNP (Bensaude, 2011). Similarly, actinomycin D induced proteasomal degradation of Esrrb (Figures 2G and 2H), and this effect was blocked by P-TEFb inhibitors DRB and flavopiridol (Figures 2I and 2J). These results demonstrate that the activation of P-TEFb by different means all converges at promoting Esrrb proteasomal degradation.

To further confirm the role of P-TEFb, we knocked down the catalytic subunit Cdk9 using shRNA. Knockdown reduced Cdk9 protein level to ∼20% of that in control cells. But unlike P-TEFb inhibitors, knockdown of Cdk9 alone did not reduce Ser-2 phosphorylation of the CTD of RNA polymerase II (Figure 2K), indicating that it was not sufficient to repress P-TEFb kinase activity in cells. This result was not surprising because most cellular pool of P-TEFb is sequestered and inactivated by 7SK snRNP (Figures 2E and 2L). We found that knockdown of Cdk9 eliminated Cdk9 in 7SK snRNP, but left the level of free P-TEFb unaffected (Figure 2L). We interpreted this result to mean that cells compensated reduced CDK9 protein level by releasing P-TEFb from 7SK snRNP in order to maintain cellular P-TEFb kinase activity. To circumvent this technical difficulty, we reasoned that knockdown of Cdk9 would require less P-TEFb inhibitors to block a P-TEFb-dependent event. We found that in control cells, 10 nM flavopiridol had no effect on cisplatin-induced Esrrb degradation, and 40 nM flavopiridol moderately blocked Esrrb degradation (lanes 1–4, Figure 2K). In contrast, with Cdk9 knockdown, 10 nM flavopiridol restored Esrrb protein level to a higher degree than 40 nM in control cells (compare lane 7 with lane 4, Figure 2K), and 40 nM fully restored Esrrb in Cdk9 knockdown cells (Figure 2K). In addition, cisplatin-induced Esrrb protein degradation in E14TG2a ES cell and F9 embryonal carcinoma cells could be restored by flavopiridol or NVP-2 treatment (Figures 2M and 2N). Similarly, UV-induced proteasomal degradation of Esrrb was blocked by P-TEFb inhibitors DRB (Figure S4), flavopiridol, or NVP-2 (Figures S9D and S9E). Taken together, we concluded that DNA damage signals activate the kinase activity of P-TEFb to induce Esrrb proteasomal degradation.

### P-TEFb directly phosphorylates Esrrb to promote its proteasomal destruction

**Figure 3:**
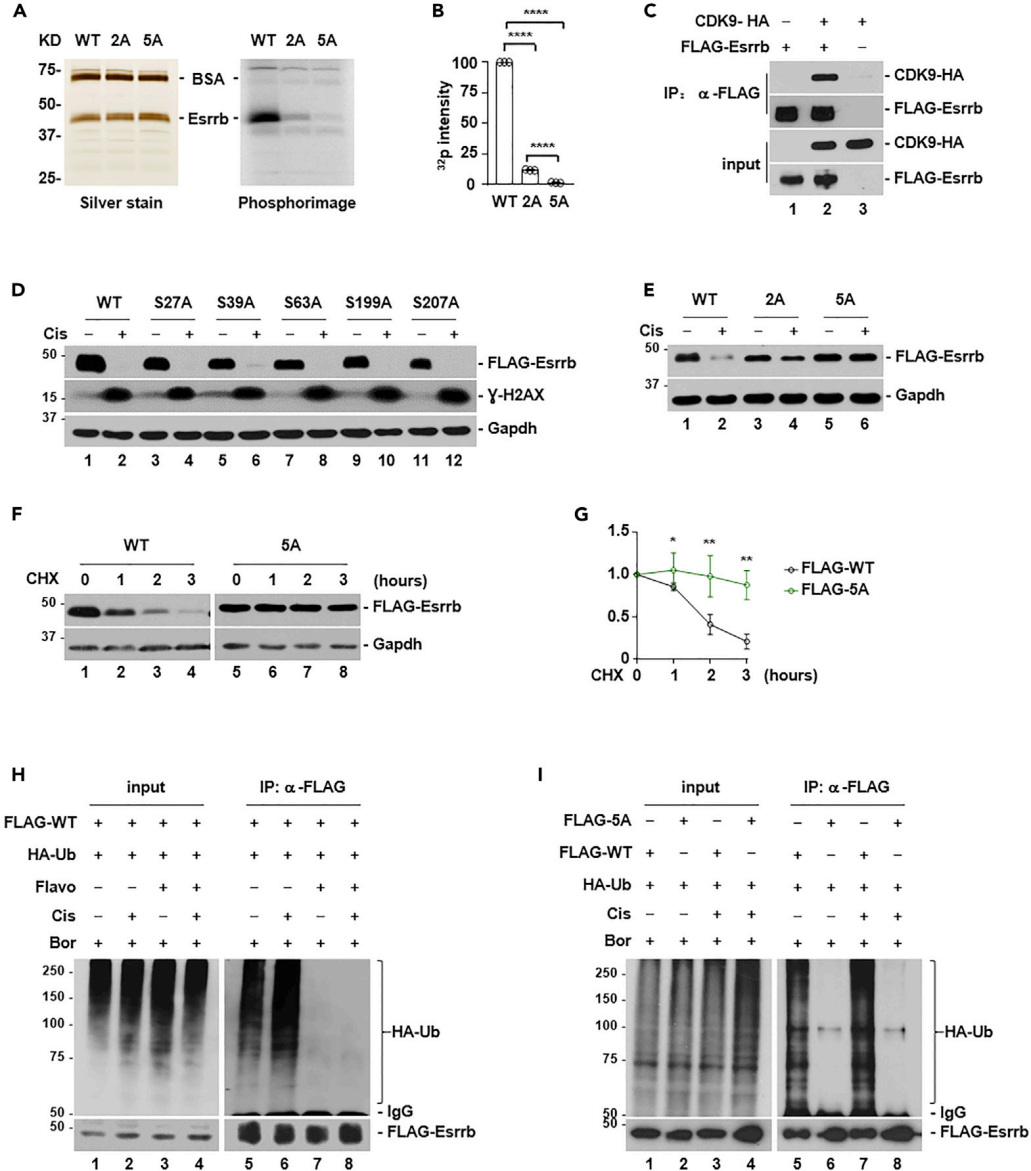
P-TEFb directly phosphorylates Esrrb protein to promote its degradation (A) P-TEFb kinase assay using recombinant wild-type (WT), 2A (S27A/S39A) or 5A (S27A/S39A/S63A/S199A/S207A) Esrrb proteins. SDS-PAGE gel was stained by silver (left panel), and then visualized by phosphorimager (right panel). BSA was used as a carrier protein. (B) Quantification of P-TEFb kinase activity in (A) (n = 3). The bar plot represents mean ± SD. ∗∗∗∗, p < 0.0001 (unpaired *t* test). (C) Analysis of the interaction between Cdk9 and Esrrb proteins by immunoprecipitation. (D) The effect of cisplatin (Cis, 5 μM) on wild-type and mutant Esrrb proteins containing individual serine residue mutated to alanine (S27A, S39A, S63A, S199A, and S207A). (E) The effect of cisplatin on wild-type and mutant Esrrb proteins containing two- or all five-serine residues mutated to alanine, 2A (S27A/S39A) or 5A (S27A/S39A/S63A/S199A/S207A), respectively. (F) Analysis of protein stability by cycloheximide (CHX, 100 μg/mL) treatment. (G) Quantification of protein stability of wild-type and 5A mutant Esrrb in (F) (n = 3). The bar plot represents mean ± SD. ∗, p < 0.05; ∗∗, p < 0.01 (unpaired *t* test). (H) Analysis of ubiquitination status of wild-type Esrrb at basal and cisplatin-treated conditions (Cis, 5 μM), in the presence or absence of P-TEFb inhibitor, flavopiridol (Flavo, 200 nM). Ub, ubiquitin. Bor, bortezomib, a proteasomal inhibitor. (I) Analysis of ubiquitination status of wild-type and 5A mutant Esrrb at basal and cisplatin-treated conditions (Cis, 5 μM). Ub, ubiquitin. Bor, bortezomib, a proteasomal inhibitor.

We hypothesized that P-TEFb might directly phosphorylate Esrrb. We carried out *in vitro* kinase assays using purified recombinant P-TEFb and Esrrb proteins. P-TEFb phosphorylates serine (S) and threonine (T) residues followed by a proline (P) residue (SP and TP sites). Esrrb protein contains five SP sites at S27, S39, S63, S199, and S207, but no TP site. Wild-type (WT) and two mutant Esrrb recombinant proteins were generated. Esrrb 2A mutant contained S27 and S39 mutated to alanine (A), and 5A contained all five serine residues mutated to alanine. The purity of all recombinant proteins was estimated to be more than 90%. Kinase reactions were resolved by SDS-PAGE gel electrophoresis, stained by silver, and then acquired by phosphorimager (Figure 3A). Wild-type Esrrb protein could be easily phosphorylated by P-TEFb. In contrast, the phosphorylation by P-TEFb was reduced down to 12% in Esrrb 2A mutant, and essentially eliminated in 5A mutant (n = 3) (Figure 3B). Consistent with Esrrb being the substrate of P-TEFb, the interaction between P-TEFb and Esrrb could be detected in cells by immunoprecipitation (Figure 3C).

Because P-TEFb promotes Esrrb degradation (Figure 2), we reasoned that these phosphorylation events might affect the protein stability of Esrrb. Cisplatin treatment induced the degradation of Esrrb proteins containing a single mutation (S27, S39, S63, S199, and S207 individually mutated to alanine) with a comparable kinetics to that of WT Esrrb protein (Figure 3D). Consistently, the protein half-life of these single mutants was similar to that of WT Esrrb. In contrast, Esrrb 2A mutant partially, and 5A mutant completely resisted cisplatin-induced degradation (Figure 3E). The protein half-life of 5A mutant was also considerably longer than that of WT Esrrb (Figures 3F and 3G). Furthermore, flavopiridol treatment blocked the ubiquitin modification on WT Esrrb under both unstressed and DNA damage conditions (Figure 3H). 5A mutant completed blocked the ubiquitin modification on Esrrb under both unstressed and DNA damage conditions without flavopiridol treatment, whereas WT Esrrb was heavily ubiquitinated (Figure 3I). These results were consistent with the notion that P-TEFb directly phosphorylates Esrrb to promote its proteasomal degradation.

### Esrrb regulates cellular sensitivity to cisplatin

Acute DNA damage undoubtably elicits highly convoluted changes in multiple cellular pathways. To disentangle this complexity, we generated Esrrb knockout cells (EKO), using CRISPR/Cas9 genomic editing technology to mimic cisplatin-induced Esrrb protein degradation. Additionally, we reconstituted EKO cells with either wild-type (EKO + WT) or 5A mutant Esrrb (EKO+5A). Reconstituted Esrrb proteins were expressed at a comparable level as that of parental cells (Figure S5A). EKO, EKO + WT, and EKO+5A cells were viable, and had indistinguishable cell cycle profiles from that of WT cells (Figure S5B). They also retained undifferentiated cellular state, indicated by comparable expression of pluripotency markers, Oct4 and Pecam1 (Figures S5A and S5C), positive alkaline phosphatase staining (Figure S5D), and repression of lineage-specific transcripts representative of differentiated germ layers (Figure S5E). In addition, transcriptomic analysis by RNA sequencing (RNA-seq) showed a high concordance of overall gene expression profiles. Focusing on 140 genes in the category of “signaling pathways regulating pluripotency of stem cells - *Mus musculus* (mouse)” (KEGG pathway: mmu04550), we found that expression levels of these genes were indistinguishable among WT, EKO, EKO-WT, and EKO-5A (Figure S5F). These results indicated that knockout of Esrrb does not compromise pluripotency, and 5A Esrrb mutant is functionally equivalent to endogenous or reconstituted WT Esrrb under homeostatic conditions.

**Figure 4:**
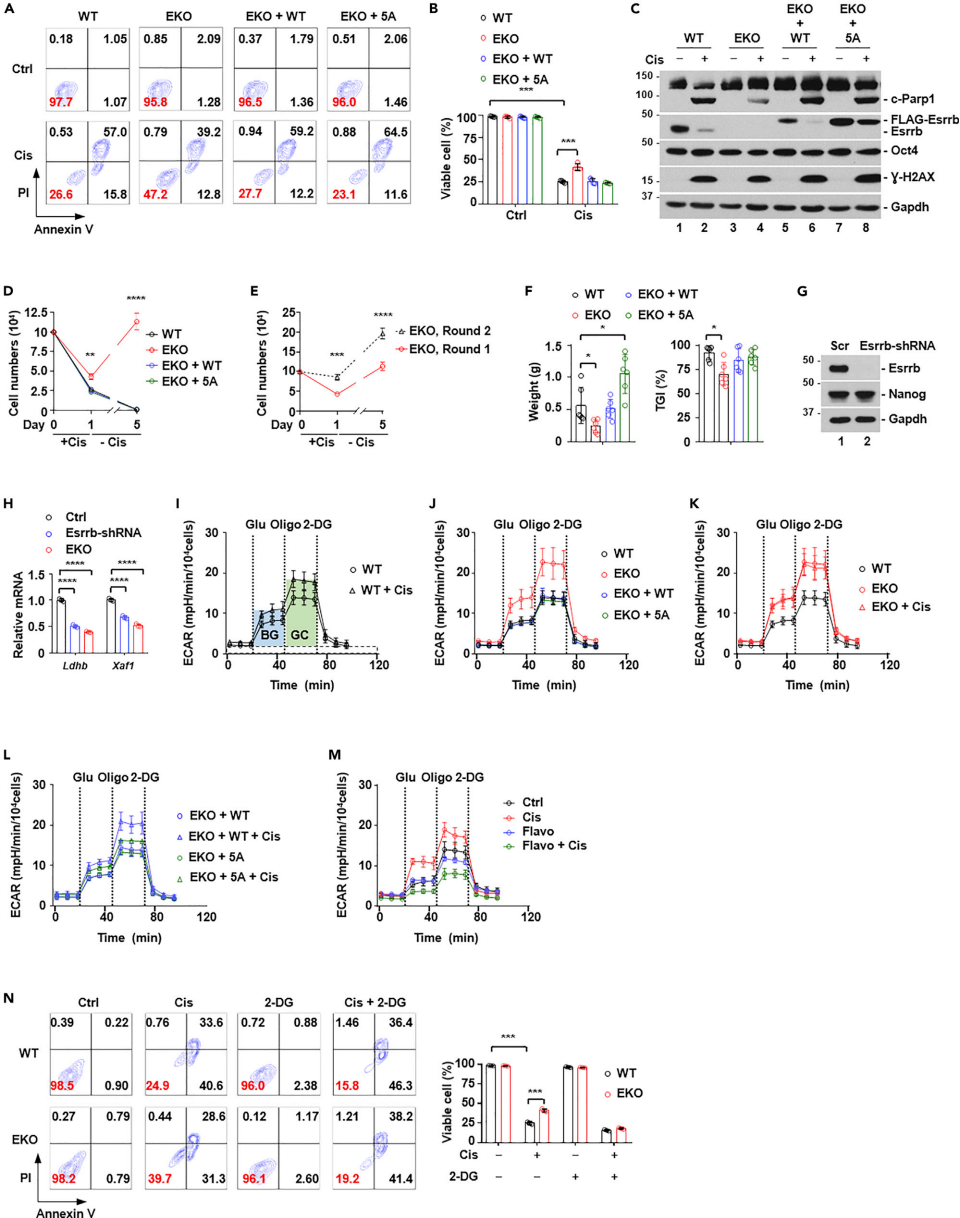
Esrrb regulates cellular sensitivity to cisplatin (A) FACS analysis of cell viability. Cells negative for both annexin V and propidium iodide (PI) were defined as live cells. Numbers denote the percentage of cells in each population, with that of live cells labeled in red. WT, wild-type cell. EKO, Esrrb knockout cell. EKO + WT, EKO cells reconstituted with wild-type Esrrb. EKO+5A, EKO cells reconstituted with Esrrb 5A mutant (S27A/S39A/S63A/S199A/S207A). Cis, cisplatin (5 μM). (B) Quantification of the percentage of live cells in (A) (n = 4). The bar plot represents mean ± SD. ∗∗∗, p < 0.001 (unpaired *t* test). (C) Western blot analyses of indicated proteins from total cell lysate of WT, EKO, EKO + WT, and EKO+5A, treated with or without cisplatin (Cis, 5 μM). Cleaved Parp1 (c-Parp1) is a marker of cell death. Oct4 is a marker of pluripotency. (D) Cell viability after one day of 5 μM cisplatin treatment (+Cis), followed by the recovery for four days in cisplatin-free (-Cis) medium. The number of viable cells for WT, EKO, EKO + WT and EKO+5A was counted at day 0, 1 and 5, and plotted (n = 3). Dot plots represent mean ± SD. ∗∗, p < 0.01; ∗∗∗∗, p < 0.0001 (unpaired *t* test). (E) Rechallenge of surviving cells from similar experiment as in (D) with cisplatin. Surviving cells after one round of cisplatin treatment as in (D) (EKO, Round 1) were treated with 5 μM cisplatin for one day (+Cis), followed by recovery for four days in cisplatin-free fresh medium (EKO, Round 2). Dot plots represent mean ± SD. ∗∗, p < 0.01; ∗∗∗∗, p < 0.0001 (unpaired *t* test). (F) Comparison of the ability of WT, EKO, EKO + WT, and EKO+5A cells to form teratoma *in vivo* (n = 6) (left panel). Comparison of the sensitivity of WT, EKO, EKO + WT, and EKO+5A cells to cisplatin *in vivo* (n = 6) (left panel). Tumor growth inhibition ratio (TGI, %) (right panel) was defined using the following formula: TGI (%) = [1 − (weight of the treated group)/(weight of the control group)] X 100 (%). The bar plot represents mean ± SD. ∗, p < 0.05 (unpaired *t* test). (G) Western blot analysis of the efficiency of Esrrb knockdown by shRNA. (H) qPCR analysis of potential Esrrb-regulated genes in Esrrb knockout (EKO) and knockdown (Esrrb-shRNA) cells. (I) Effect of cisplatin on the glycolytic rate. Real-time extracellular acidification rate (ECAR) was measured at indicated time points by Seahorse extracellular flux analyzer (n = 3). Glucose (Gluc), oligomycin (Oligo), and 2-Deoxyglucose (2-DG) were injected at the indicated time points. The height of the area shaded by light-blue denotes the degree of basal glycolytic rate (BG). The height of the area shaded by light-green denotes the degree of maximal glycolytic capacity (GC). (J) Comparison of the ECAR between WT, EKO, EKO + WT, and EKO+5A cells (n = 3). ECAR for WT cells was the same as in (I). (K) Comparison of the ECAR in EKO cells with or without cisplatin (n = 3). ECAR for WT cells was the same as in (I and J), and that for EKO cells was the same as in (J). (L) Comparison of the ECAR in EKO + WT and EKO+5A cells with or without cisplatin (n = 3). (M) Comparison of the ECAR with or without cisplatin, in the presence or absence of chemical inhibitors of P-TEFb, flavopiridol (Flavo, 200 nM). (N) Effect of cisplatin-induced glycolysis on cell viability. Cells negative for both annexin V and PI by FACS analysis were defined as live cells. Numbers denote the percentage of cells in each population, with that of live cells labeled in red (left panel). Quantification of the percentage of live cells (right panel) (n = 3). 2-DG is a chemical inhibitor of glycolysis. The bar plot represents mean ± SD. ∗∗∗, p < 0.001 (unpaired *t* test).

We analyzed the sensitivity of these cells to cisplatin. After cisplatin treatment for 24 h, the percentage of viable cells (defined by the cell population negatively stained for both annexin V and propidium iodide) was indistinguishable between WT and EKO + WT. EKO+5A had a small but highly reproducible decrease in viability compared to WT and EKO + WT cells. In contrast, EKO cells showed a 2-fold increase in viability (Figures 4A and 4B). Consistently, the cleavage of Parp1 by apoptotic caspases was less prominent in EKO cells (Figure 4C). Of note, Oct4 protein level was not altered at 24 h after cisplatin treatment (Figure 4C), indicating that increased resistance was not due to cellular differentiation. Furthermore, we removed cisplatin after 24 h, replenished remaining cells with fresh culture medium without cisplatin, and cultured for additional four days. This treatment procedure mimics the clinical scenario because cisplatin is largely eliminated from the human body after 24 h (Breglio et al., 2017). Interestingly, essentially no cells were left for WT, EKO + WT, and EKO+5A at day 5, consistent with the fact that embryonal stem cells are exquisitely sensitive to cisplatin. In contrast, a significant amount of EKO cells were viable and proliferative (Figure 4D). Thus, the remaining, apparently alive cells from WT, EKO + WT, and EKO+5A genetic background after 24-h cisplatin treatment (Figures 4A and 4B) were actually on the path to cell death. To further ascertain that EKO cells were truly resistant, we repeated the experiment as in Figure 4D, and collected surviving EKO cells (EKO, Round 1, Figure 4E). We then treated these cells with cisplatin for 1 day, followed by culturing in cisplatin-free medium for another 4 days (EKO, Round 2, Figure 4E). EKO cells survived from the 1st round of cisplatin treatment demonstrated higher resistance to cisplatin than treatment naive EKO cells (Figure 4E). To examine cisplatin sensitivity *in vivo*, we used standard teratoma formation assay (Nelakanti et al., 2015). WT, EKO, EKO + WT, or EKO+5A cells were subcutaneously inoculated in immunodeficient NOG-dKO mice (Yaguchi et al., 2018). Consistent with the fact that Esrrb promotes stem cell identity, we found that EKO+5A had higher, whereas EKO exhibited lower malignant potential than WT and EKO + WT cells (Figure 4F, left panel). However, the tumor growth inhibition ratio (TGI) of cisplatin was close to 100% in WT, EKO + WT, and EKO+5A cells, but only around 70% in EKO cells (Figure 4F, right panel). These results indicated that reduced Esrrb activity promotes cisplatin resistance.

Because Esrrb functions as a transcription factor, we reasoned that differentially expressed genes might explain the difference in cisplatin sensitivity. Overall, among 17,101 genes well expressed in WT or EKO cells (transcripts per kilobase of exon model per million mapped reads, TPM >1), 756 genes exhibited a 2-fold difference (296 upregulated and 460 downregulated; p < 0.05). The expression of 82.3% and 81.5% of these differentially expressed genes in EKO could be rescued by re-introduction of WT or 5A Esrrb in EKO cells, respectively (Figure S5G). We reasoned that these genes were likely regulated by Esrrb directly or indirectly. Furthermore, we queried two recently published anti-Esrrb ChIP-seq datasets to infer genes potentially regulated by Esrrb (Festuccia et al., 2016, 2019). We considered a gene as a potential Esrrb target if Esrrb is bound within 2,000 bp of its annotated transcriptional start site. We noticed a moderate overlap between these datasets (Figure S6A). Nevertheless, we were able to identify several shared targets by ChIP-seq and our RNA-seq analyses (Figure S6B). We confirmed the changes in several genes by qPCR. Additionally, we used shRNA to knockdown Esrrb (Figure 4G), followed by qPCR analysis of these genes. In general, we found that the trend of changes in gene expression was similar between Esrrb knockdown and knockout (Figures 4H and S6D). Interestingly, these analyses revealed that the expression of lactate dehydrogenase B (Ldhb) was reduced by both Esrrb knockdown and knockout (Figure 4H). Ldhb catalyzes the conversion of lactate to pyruvate, and thus tips the balance in favor of mitochondria oxidative phosphorylation over aerobic glycolysis (Brisson et al., 2016). The major function of aerobic glycolysis is to replenish biomass such as nucleotides, amino acids, and lipids (Lunt and Vander Heiden, 2011; Vander Heiden et al., 2009), all of which are damaged by cisplatin (Galluzzi et al., 2014). We reasoned that reduced Ldhb expression might render a more glycolytic intracellular environment to compensate damaging effects of cisplatin. Using Seahorse glycolytic rate assay (Divakaruni et al., 2014), we found that cisplatin treatment enhanced both basal glycolytic rate and the maximal glycolytic capacity in WT cells (Figure 4I). Of note, glycolytic rate assay was carried out within 10 h of cisplatin treatment, and cells were negative by PI staining at this stage. Thus, cisplatin-induced increase in glycolytic rate preceded cell death, consistent with the protective role of glycolysis. Remarkably, both basal glycolytic rate and the maximal glycolytic capacity were increased in untreated EKO cells, to a comparable degree as cisplatin-treated WT cells (Figure 4J). Cisplatin treatment did not further enhance basal glycolytic rate or the maximal glycolytic capacity in EKO cells (Figure 4K). Consistently, EKO+5A cells showed only slightly increased glycolytic rate after cisplatin treatment (Figure 4L). We carried out glycolytic rate assay in WT cells treated with cisplatin and flavopiridol (Figure 4M). Consistently, cisplatin treatment alone increased the glycolytic rate. Flavopiridol treatment alone slightly decreased the glycolytic rate. In contrast, flavopiridol treatment fully blocked cisplatin-induced glycolytic rate. This is consistent with the notion that P-TEFb-induced Esrrb degradation promotes glycolysis. We also noticed that the effect of flavopiridol was greater than Esrrb 5A mutant to block cisplatin-induced glycolysis (Figure 4L), highlighting that glycolysis and cellular responses to cisplatin are highly complex. To confirm the protective role of enhanced glycolysis, we treated EKO cells with 2-deoxyglucose (2-DG), a widely used chemical inhibitor of glycolysis. Indeed, 2-DG could restore cisplatin sensitivity in EKO cells to a similar degree as that in WT cells (Figure 4N). Lastly, we considered the possibility that loss of Esrrb might alter mitochondrial functionality to account for enhanced glycolysis and cisplatin resistance. We used the BH3 profiling assay to functionally probe mitochondrial priming, that is, a cell’s “readiness” for apoptosis (Fraser et al., 2019). Mitochondrial priming has been shown to be positively correlated with chemosensitivity of patients in multiple cancers (Potter and Letai, 2016). We also measured mitochondrial content, membrane potential, and reactive oxygen species. In all cases, we found that these parameters were indistinguishable among WT, EKO, EKO + WT, and EKO+5A cells (Figure S7). Thus, increased cisplatin resistance in EKO cells was unlikely caused by the perturbation of the mitochondrial apoptotic threshold. Taken together, these results indicate that P-TEFb-dependent degradation of Esrrb protein is a cellular defensive mechanism to counteract the damaging effects of cisplatin.

### Inhibition of P-TEFb abolishes cisplatin-induced cell death

**Figure 5:**
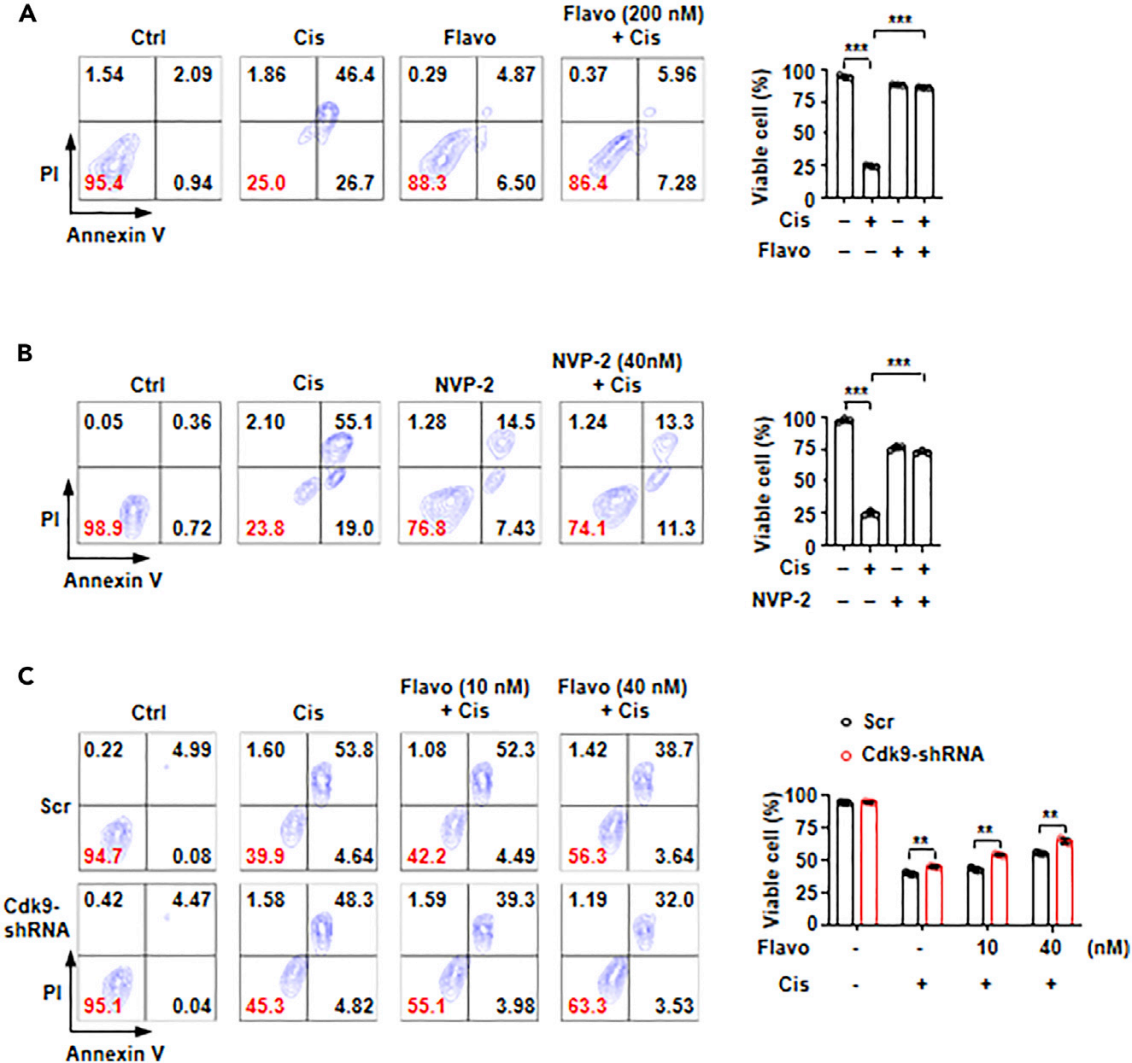
P-TEFb inhibitors abolish cisplatin-induced cell death (A) Effect of P-TEFb inhibitor, flavopiridol, on cisplatin-induced cell death. Cells negative for both annexin V and propidium iodide (PI) by FACS analysis were defined as live cells. Numbers denote the percentage of cells in each population, with that of live cells labeled in red. Cells were either untreated (Ctrl), treated with cisplatin (Cis, 5 μM) or flavopiridol (Flavo, 200 nM) individually, or in combination (Cis + Flavo) (left panel). Quantification of the percentage of live cells (right panel) (n = 3). The bar plot represents mean ± SD. ∗∗∗, p < 0.001 (unpaired *t* test). (B) Similar experiment as in (A) except that NVP-2 was used to inhibit P-TEFb kinase activity (n = 3). The bar plot represents mean ± SD. ∗∗∗, p < 0.001 (unpaired *t* test). (C) The effect of Cdk9 knockdown by small-hairpin RNA (shRNA) on cisplatin-induced cell death. The bar plot represents mean ± SD. ∗∗, p < 0.01 (unpaired *t* test).

There are growing interests in developing pharmaceutical inhibitors of P-TEFb to treat cancer, under the assumption that fast proliferating cells would require higher global transcriptional output by RNA polymerase II (Wu et al., 2020). Because P-TEFb-induced Esrrb degradation favors cell survival, we anticipated that chemical inhibition of P-TEFb would act synergistically with cisplatin to promote cell death. Surprisingly, we found that either pre- or co-treatment with small-molecule inhibitors of P-TEFb, flavopiridol, or NVP-2, largely blocked cisplatin-induced cell death (Figures 5A and 5B). Both P-TEFb inhibitors alone did not induce obvious cell cycle arrest. In fact, cells continued to proliferate for days in the presence of P-TEFb inhibitors. To show on-target effect of flavopiridol on CDK9, we used 10 nM or 40 nM in combination with or without CDK9 knockdown. Indeed, knockdown of CDK9 enhanced the effect of flavopiridol to block cisplatin-induced cell death (Figure 5C). In addition, P-TEFb inhibitor flavopiridol blocked UV-induced cell death (Figure S8). These results indicated that acute DNA damage-induced death programs also require P-TEFb kinase activity.

**Figure 6:**
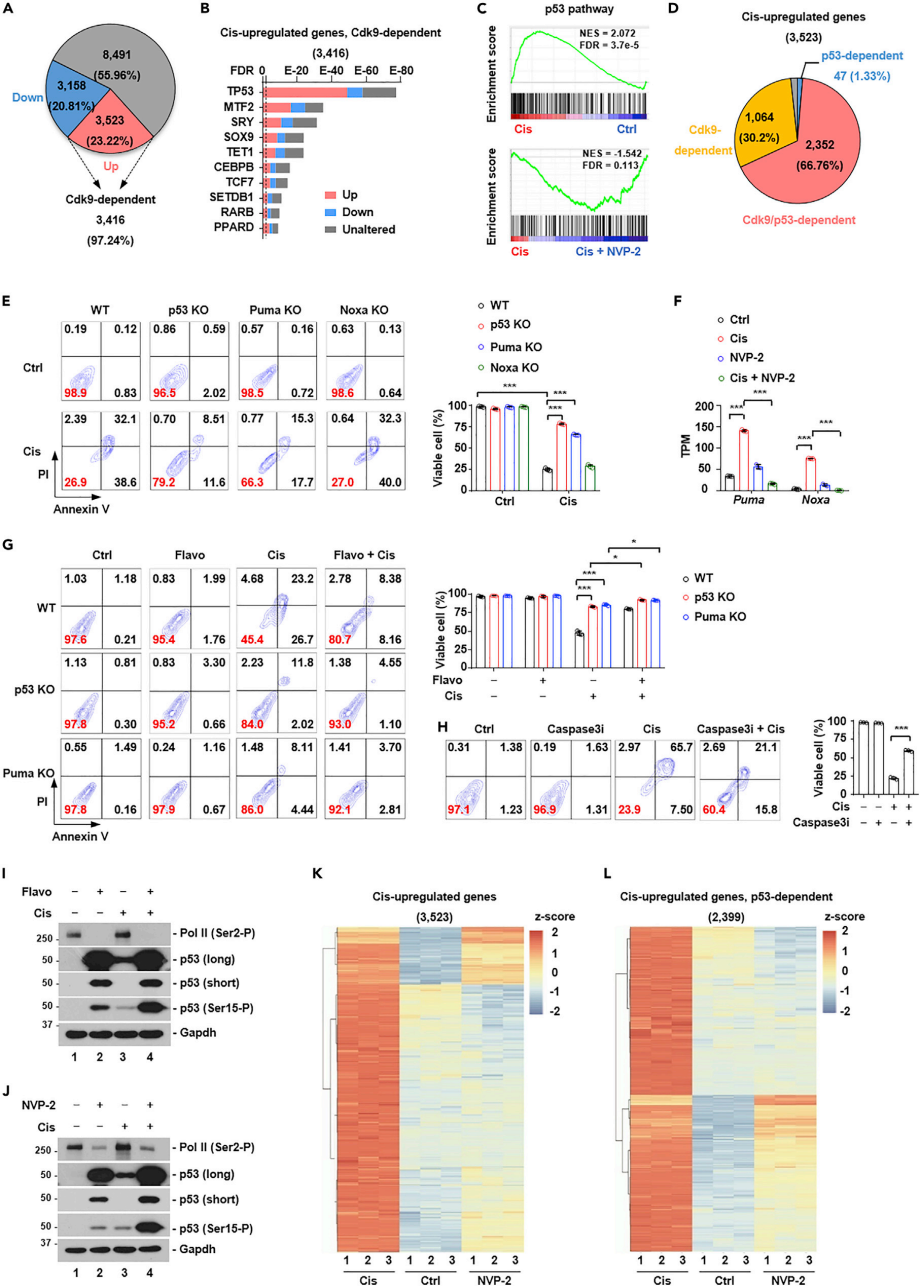
P-TEFb is a global regulator of cisplatin-induced transcriptional reprogram (A) Pie chart presentation of the dependency of cisplatin-regulated genes on P-TEFb by RNA sequencing (RNA-seq). The percentage inside the pie chart denotes the percentage of genes unaltered (55.96%), downregulated (20.81%) or upregulated (23.22%) by cisplatin. The percentage outside the pie chart denotes the percentage of genes upregulated by cisplatin in a P-TEFb-dependent manner (97.24%). (B) Transcription factor enrichment analysis of cisplatin-induced, P-TEFb-dependent genes by ChIP-X Enrichment Analysis 3 (ChEA3) (Keenan et al., 2019). Top ten transcription factors predicted are listed and ranked by false discovery rate (FDR). Vertical dashed line denotes FDR = 0.01. Out of 982 gene promoters bound by p53 in ChEA3, 708 were expressed in cisplatin-treated R1/E cells (TPM > 1). (C) Gene set enrichment analysis (GSEA) of the effect of P-TEFb inhibitor NVP-2 on cisplatin-induced pathways. p53 transcriptional program was shown as an example. NES, normalized enrichment score. FDR, false discovery rate. (D) Pie chart presentation of the dependency of cisplatin-regulated genes on P-TEFb and p53. (E) FACS analysis of the sensitivity of wild-type (WT), p53-, Puma-, and Noxa-knockout (KO) cells to cisplatin (Cis, 5 μM). Cells negative for both annexin V and propidium iodide (PI) were defined as live cells. Numbers denote the percentage of cells in each population, with that of live cells labeled in red (left panel). Quantification of the percentage of live cells (right panel) (n = 3). The bar plot represents mean ± SD. ∗∗∗, p < 0.001 (unpaired *t* test). (F) The effect of P-TEFb inhibitor NVP-2 (20 nM) on cisplatin-induced expression of Puma and Noxa (n = 3). The bar plot represents mean ± SD. ∗∗∗, p < 0.001 (unpaired *t* test). (G) The effect of P-TEFb inhibitor flavopiridol (Flavo, 200 nM) on cisplatin-induced cell death in p53-or Puma-knockout (KO) cells. Quantification of the percentage of live cells (right panel) (n = 3). The bar plot represents mean ± SD. ∗, p < 0.05, ∗∗∗, p < 0.001 (unpaired *t* test). (H) The effect of Caspase-3 inhibitor on cisplatin-induced cell death. Cells negative for both annexin V and propidium iodide (PI) by FACS analysis were defined as live cells. Numbers denote the percentage of cells in each population, with that of live cells labeled in red. Cells were either untreated (Ctrl), treated with cisplatin (Cis, 5 μM) or Caspase-3 inhibitor (Caspase-3i, 100 μM) individually, or in combination (Caspase-3i + Cis) (left panel). Quantification of the percentage of live cells (right panel) (n = 3). The bar plot represents mean ± SD. ∗∗∗, p < 0.001 (unpaired *t* test). (I and J) The effect of P-TEFb inhibitors flavopiridol (Flavo, 200 nM) (I) and NVP-2 (20 nM) (H) on p53 expression. Long, long exposure. Short, short exposure. (K) Heatmap presentation of cisplatin-induced genes. Most of these genes were not induced by NVP-2 treatment (n = 3). (L) Heatmap presentation of cisplatin-induced p53 transcriptional program. Most of these genes were not induced by NVP-2 treatment (n = 3).

Because P-TEFb regulates gene transcription, we carried out transcriptomic analysis by RNA-seq of untreated cells, cells treated with cisplatin, NVP-2 individually or in combination. Using TPM >1 as cut-off (15,172 genes), we found that compared to untreated cells, 3,523 genes were upregulated and 3,158 genes downregulated (fold change >2) in cisplatin-treated cells (Figure 6A). We focused on the characterization of cisplatin-upregulated genes because the well-known function of P-TEFb is to stimulate gene expression. Co-treatment with NVP-2 reduced 97.24% of cisplatin-induced genes by more than 2-fold (3,416/3,523 genes) (Figure 6A). We reasoned that some of these genes might contribute to the effect of P-TEFb inhibitors to block cisplatin-induced death. In this regard, activating transcription factor 3 (Atf3) stood out as a strong candidate. Our RNA-seq datasets indicated that Atf3 was barely transcribed under homeostatic conditions. Treatment with cisplatin greatly upregulated Atf3 expression and co-treatment with NVP-2 reduced Atf3 expression back to the basal level at both the mRNA and protein levels (Figures S9A and S9B). Similarly, Atf3 was induced by UV treatment, and flavopiridol or NVP-2 completely blocked UV-induced Atf3 expression (Figures S9C, S9D, and S9E). Atf3 is known to be induced by diverse stress signals (Ku and Cheng, 2020), although its role in chemosensitivity is unknown. We decided to investigate whether Atf3 was required to mediate the effect of P-TEFb inhibitors on cisplatin-induced cell death. We generated several Atf3 knockout cell lines, and confirmed that cisplatin no longer induced detectable Atf3 protein expression in these cells (Figure S9F). Surprisingly, knockout of Atf3 had little effect on cisplatin-induced cell death (Figure S9G). Thus, although Atf3 is activated by cisplatin in a P-TEFb-dependent manner, it does not contribute to cisplatin-induced cell death.

### P-TEFb is required for cisplatin-induced p53 transcriptional program

To understand how P-TEFb inhibitors blocked cisplatin-induced cell death, we queried genes induced by cisplatin in a P-TEFb-dependent manner (3,416 genes, Figure 6A). We used ChEA3 to analyze whether these genes were regulated by shared transcription factors (Keenan et al., 2019). This analysis indicated that many genes were potential transcriptional targets of tumor suppressor protein p53 (Figure 6B). In addition, gene set enrichment analysis (GSEA) using the hallmark gene set collection in the Molecular Signatures Database (MSigDB) (Liberzon et al., 2015) demonstrated that chemical inhibition of P-TEFb downregulated cisplatin-induced p53 transcriptional program (Figure 6C). This result was unexpected because the transcription of p53 target genes was previously thought to be independent of P-TEFb (Gomes et al., 2006). To examine the relationship between p53 and P-TEFb, we generated p53 knockout cell lines. In these cells, UV, cisplatin, or MG132 treatment was no longer able to induce p53 protein expression (Figure S9H and S9J). We treated wild-type and p53 knockout cells with cisplatin, NVP-2, or both, and carried out transcriptomic analysis by RNA-seq. Focusing on 3,523 genes induced by cisplatin (Figure 6A), we found that 1,064 genes (30.2%) were dependent on P-TEFb but not p53, 2,352 genes (66.76%) dependent on both P-TEFb and p53, and only 47 genes (1.33%) dependent on p53 but not P-TEFb (Figure 6D). Thus, P-TEFb kinase activity is required for the transcription of a substantial portion of p53 target genes, and is a global regulator of cisplatin-induced transcriptional program. As an illustrative example, we found that UV-induced Atf3 protein expression was completely abolished in p53 knockout cells (Figure S9I). Thus, Atf3 is a p53 target gene induced by DNA damage in a P-TEFb-dependent manner (Figure S9). Furthermore, we carried out similar analysis with UV treatment, and found P-TEFb was required for a large portion of p53-dependent transcription (Figure S10). Thus, P-TEFb is a global regulator of acute DNA damage-induced transcriptional program including that of p53.

We asked whether P-TEFb inhibitors counteracted cisplatin-induced cell death via the regulation of p53 transcriptional program. We found that p53 knockout cells largely resisted cisplatin-induced cell death (Figure 6E), indicating the involvement of p53 target genes. Focusing on several hundred p53 target genes compiled by two recent meta-analysis studies (Andrysik et al., 2017; Fischer, 2017), we found that Bbc3 (commonly known as Puma) and Pmaip1 (commonly known as Noxa) were induced by cisplatin, and this induction was largely abolished by NVP-2 (Figure 6F). Both Puma and Noxa are known to induce cell death. We found that knockout of Puma, but not Noxa, largely abolished cisplatin-induced cell death, to a similar degree as that of p53 (Figure 6E), and flavopiridol treatment further blocked cisplatin-induced cell death in p53 knockout or Puma knockout cells (Figure 6G). Consistent with the fact that p53-Puma axis induces caspase-dependent cell death, treatment with caspase-3 inhibitor (caspase-3i) also blocked cisplatin-induced cell death (Figure 6H). Notably, flavopiridol or NVP-2 treatment alone strongly induced p53 protein expression, to a greater degree than cisplatin (Figures 6I and 6J). The level of Ser15 phosphorylation, an indicator of p53 transcriptional activity, was also stimulated (Figures 6I and 6J). However, a majority of cisplatin-induced genes (Figures 6A, 3,523 genes) were not activated by NVP-2 treatment alone (Figure 6K). Similarly, cisplatin-induced genes that are dependent on p53 (Figure 6D, 2,399 genes) were largely not induced by NVP-2 treatment alone (Figure 6L). Thus, chemical inhibitors of P-TEFb increase p53 protein level, but simultaneously blocks p53-depdent transcriptional program. The mechanism how P-TEFb inhibitors induce p53 stabilization remains to be elucidated. Nevertheless, these results explain why p53-dependent cell death is blocked despite massive p53 protein accumulation, and consistent with the idea that P-TEFb inhibitors blocked p53-dependent transcriptional program to counteract cisplatin-induced cell death.

## Discussion

Cisplatin is a popular chemotherapy drug for the treatment of a variety of human cancers such as those of testis, ovary, and lung. However, for most tumor types, primary and acquired resistance to cisplatin presents a serious clinical challenge that ultimately leads to patient demise (Rottenberg et al., 2021). One notable exception is testicular germ cell tumor (TGCT). A high cure rate is achieved in TGCTs due to their exceptional sensitivity to cisplatin. This sensitivity is thought to be associated with their embryonal stem cell-like, undifferentiated cellular state, although the underlying molecular mechanisms remain incompletely understood (Oosterhuis and Looijenga, 2019). Of note, recent study established that embryonic stem cells can exist in different pluripotent states (termed naive and primed states), expressing master pluripotency transcription factors, OCT4 and SOX2, at similar levels (Weinberger et al., 2016). In contrast, ESRRB is highly expressed in naive but not the primed pluripotency state, and can promote the conversion of embryonic stem cells from primed to naive state (Adachi et al., 2018). Thus, downregulation of ESRRB marks the earliest event in pluripotent cells on the path toward differentiation (Festuccia et al., 2018). We show that DNA damage triggers ESRRB protein degradation, and reduced ESRRB activity seems to enhance aerobic glycolysis to promote cell survival. Cisplatin damages biomolecules including lipids, nucleic acids, and proteins. Because enhanced glycolysis promotes anabolism to synthesize biomolecules, we suspect that this is an adaptive response of cells trying to compensate the damaging effect of cisplatin on these biomolecules. DNA damage-induced glycolysis is also recently observed in cancer cells (Li et al., 2018). In addition to ESRRB, we find that DNA damage induces the proteasomal degradation of NANOG protein, although none of chemical inhibitors we tested could block this degradation (except for proteasome inhibitors). Of note, both ESRRB and NANOG are known to promote self-renewal of embryonic stem cells. Thus, DNA damage induces cell differentiation, followed by subsequent p53-dependent cell death. We find that small-molecule inhibitors of P-TEFb can largely block cisplatin- and UV-induced ESRRB protein degradation, but have no effect on NANOG protein degradation. Strikingly, we find that P-TEFb inhibitors prevent cisplatin-induced cell differentiation and cell death. Notably, ESRRB can functionally substitute NANOG in pluripotency maintenance (Zhang et al., 2018). This explains why P-TEFb inhibitors can sustain self-renewal in the presence of cisplatin despite reduced NANOG. Taken together, these results establish ESRRB as a molecular link between pluripotent cellular state and cisplatin sensitivity.

TGCT is cured with cisplatin at a high rate. Unfortunately, the life quality of longer survivorship is compromised by cisplatin-induced severe ototoxicity in many patients (Freyer et al., 2019). Ototoxicity is likely to be caused by the accumulation of cisplatin in the stria vascularis region of the cochlea in human inner ear (Breglio et al., 2017). However, little is known how cisplatin compromises inner ear function. It is intriguing that ESRRB is expressed exactly in this anatomic location in the inner ear, and seems to be important to maintain inner ear functionality (Chen and Nathans, 2007; Collin et al., 2008). Our results demonstrate that cisplatin induces the degradation of ESRRB protein. It is tempting to speculate that ESRRB protein degradation may at least partly contribute to cisplatin-induced ototoxicity. This hypothesis can be tested by generating knockin mice containing 5A ESRRB mutant protein. We find that 5A mutant is similar to the wild-type ESRRB in transcribing its target genes, but largely resists cisplatin-induced protein degradation. We also demonstrate that pharmaceutical P-TEFb inhibitors can fully block cisplatin-induced ESRRB protein degradation. Recently, there has been growing interests in developing specific P-TEFb inhibitors as anticancer therapeutics (Wu et al., 2020). This may provide a translational opportunity to examine whether these drugs can ameliorate cisplatin-induced ototoxicity in animal models as well as clinical trials. To avoid any potential interference with cisplatin (discussed below), local drug delivery strategies such as microneedle array infusion devices can be used to apply P-TEFb inhibitors directly to the inner ear (Freyer et al., 2020).

Our results demonstrate that P-TEFb is a previously underappreciated regulator of the transcriptional response to DNA damage. Under physiological conditions, P-TEFb is well known to promote RNA polymerase II transcription via direct phosphorylation of its largest subunit (Zhou et al., 2012). Thus, it is generally assumed that pharmaceutical inhibitors of P-TEFb will have anticancer effects (Wu et al., 2020). However, molecular determinants of cellular sensitivity to P-TEFb inhibitors remain largely unknown. Another layer of complexity is that most cellular pool of P-TEFb is sequestered and inactivated by 7SK snRNP. It has known for two decades that UV treatment rapidly increases P-TEFb activity by releasing it from inhibitory 7SK snRNP (Nguyen et al., 2001), but the implication of this regulation for cellular DNA damage response is not clear. We demonstrate that acute DNA damage-induced transcriptional changes, either by cisplatin or UV, largely depend on P-TEFb. This indicates that P-TEFb may modulate activities of multiple transcription factors beyond its well-established substrate, RNA polymerase II. Indeed, we find that cisplatin, UV, and doxorubicin all activate P-TEFb to directly phosphorylate transcription factor ESRRB and induce its proteasomal degradation. On the other hand, we show that P-TEFb is required to transcriptionally upregulate ATF3, a transcription factor associated with a variety of stress conditions (Ku and Cheng, 2020). Although p53 transcriptional program was previously thought to be independent of P-TEFb (Gomes et al., 2006), we unexpectedly find that ATF3 as well as a large portion of p53 target genes depends on P-TEFb for their transcriptional activation. Chemical inhibition of P-TEFb seems to be sufficient to blunt p53-induced cellular responses. Cell fate, whether during normal development or under stressed conditions, is determined by the complex interplay between master transcription factors (Graf and Enver, 2009; Stadhouders et al., 2019). In case of chemosensitivity, our results demonstrates that P-TEFb-dependent p53 program to promote cell death outweighs P-TEFb-dependent ESRRB degradation to promote cell survival (Figure S12). Thus, the outcome of P-TEFb inhibitors may be dictated by different transcriptional dependency of diverse cell types under various conditions.

Several recent studies also examined the relationship between P-TEFb and UV-induced cell death. Studniarek et al. showed that knockout of 7SK snRNA (thus higher P-TEFb activity) in HAP1 cells rendered them more sensitive to UV irradiation (Studniarek et al., 2021). This result is consistent with our findings that chemical inhibition of P-TEFb promotes cell survival under DNA damage conditions. In another study, Bugai et al. found that flavopiridol treatment blocked p53 transcriptional program induced by 4-nitroquinoline 1-oxide (4-NQO, a UV radiation-mimetic chemical) (Bugai et al., 2019), in agreement with our results. However, they also found that flavopiridol collaborated with 4-NQO to promote cell death (Bugai et al., 2019). Considering that p53 is a major determinant of DNA damage-induced cell death, it is unclear how inactivating p53 function by flavopiridol could potentiate the function of 4-NQO. Further studies are needed to clarify this discrepancy.

In summary, our results demonstrate that P-TEFb is integral to the DNA damage response by regulating activities of multiple transcription factors including p53. Notably, recent studies demonstrate an oncogenic function of wild-type p53 and its target gene PUMA in liver cancer progression (Kim et al., 2019), suggesting that P-TEFb inhibitors may exhibit clinical benefits in this unique context. However, in tumors containing wild-type p53, P-TEFb inhibitors may antagonize cytotoxic effects of cisplatin. Lastly, because p53 pathway is inactivated in many cancers (Levine, 2020), the therapeutical effects of P-TEFb inhibitors as single agent or in combination with other drugs warrant further mechanistic investigation.

### Limitations of the study

The present study demonstrates that P-TEFb is integral to the DNA damage response to regulate cisplatin sensitivity in ES cells. ES cells are considered as normal counterparts of TGCTs, and the origin of the exquisite cisplatin sensitivity of TGCTs. Nevertheless, mutations in oncogenic and tumor-suppressive genes in TGCTs may affect DNA damage response. Indeed, among cisplatin-resistant TGCTs, 21.3% have mutations to inactivate p53 function, but the mechanisms remain undefined in the remaining ∼80% cases (Bagrodia et al., 2016). Future studies are needed to identify and address how defects in other genes and pathways affect cisplatin sensitivity in TGCTs. The present study demonstrates that chemical inhibitors of P-TEFb largely blocked cisplatin- or UV-induced cells death. Future studies are needed to extend this observation in other cancer types, considering that many tumors, such as high-grade serous ovarian carcinoma, have inactivated p53 but still respond to cisplatin-induced cell death. Lastly, the mechanism of how chemical inhibitors of P-TEFb induce p53 protein stabilization is still poorly understood. One recent study suggested that P-TEFb inhibitors reduce MDM4 expression to stabilize p53. However, the expression of MDM2, the major negative regulator of p53 stability, is not affected (Stetkova et al., 2020). Although the present study demonstrates that p53-induced transcriptional program is largely blocked by chemical inhibitors of P-TEFb, several p53 target genes are unaffected. More studies are needed to clarify these issues in order to provide rationales and patient stratification criteria to test the efficacy of chemical inhibitors of P-TEFb in clinical trials.

## STAR★Methods

### Key resources table

**Table:** 

REAGENT or RESOURCE	SOURCE	IDENTIFIER
**Antibodies**		

ESRRB	R&D	Cat# PP-H6705-00
FLAG M2	Sigma	Cat# A8562
RNA Pol II	Covance	Cat# 8WG16, MPY-127R
p-RNA Pol II (Ser2)	Covance	Cat# H5, MPY-129R
HA	Santa Cruz	Cat# sc-7392
Cdk9	Santa Cruz	Cat# sc-484
CDC25a	Santa Cruz	Cat# sc-7389
Chk1	Santa Cruz	Cat# sc-8408
Oct4	Santa Cruz	Cat# sc-9081
p53	Santa Cruz	Cat# sc-393031
Nanog	Bethyl	Cat# A300-397A
Ɣ-H2AX	Abcam	Cat# ab2893
p-RB (Ser807+Ser811)	Abcam	Cat# ab277774
Gapdh	KangChen	Cat# KC-5G5

**Chemicals, peptides, and recombinant proteins**		

Cisplatin	MCE	Cat# HY17394
Doxorubicin	Sigma	Cat# D1515
DRB	Sigma	Cat# D1916, 25
2-deoxyglucose	Sigma	Cat# D8375
Bortezomib	MCE	Cat# HY10227
Oligomycin	MCE	Cat# HY-N6782
Chloroquine	Selleck	Cat# S6999
KU-60019	Selleck	Cat# S1570
VE821	Selleck	Cat# S8007
NU7441	Selleck	Cat# S2638
SB203508	Selleck	Cat# S1076
AZD7762	Selleck	Cat# S1532
Flavopiridol	Selleck	Cat# S1230
NVP-2	Selleck	Cat# S8981
THZ1	Selleck	Cat# S7549
THZ531	Selleck	Cat# S6595
RO-3306	Selleck	Cat# S7747
Palbociclib	Selleck	Cat# S4482

**Critical commercial assays**		

cDNA Reverse Transcription Kit	Invitrogen	Cat# 28025021
SYBR green Mastermix	Biorad	Cat# 1725271
BCA Protein Assay Kit	ThermoFisher	Cat# 23225
FITC Annexin V Apoptosis Detection Kit	BD Biosciences	Cat# 556547
488 EdU Click Proliferation Kit	BD Biosciences	Cat# 565455
Seahorse XF Glycolysis Stress Test Kit	Agilent	Cat# 103020-100
MitoSOX red	ThermoFisher	Cat# M36008
MitoTracker Green FM	ThermoFisher	Cat# M7514
MitoTracker Red CM-H2XRos	ThermoFisher	Cat# M7513

**Deposited data**		

RNA-seq data	This paper	PRJNA827641

**Experimental models: Cell lines**		

R1/E	ATCC	Cat# SCRC-1036
E14Tg2a	ATCC	Cat# CRL-1821
F9	ATCC	Cat# CRL-1720
HEK293T	ATCC	Cat# CRL-3216
HCT116	ATCC	Cat# CCL-247
R1/E Esrrb knockout cell line	This paper	N/A
R1/E Esrrb knockout transfected Esrrb WT cell line	This paper	N/A
R1/E Esrrb knockout transfected Esrrb 5A cell line	This paper	N/A

**Experimental models: Organisms/strains**		

Mouse: NOG-dKO	Charles River	Cat# 411

**Oligonucleotides**		

qPCR primers (Table S1)	This paper	N/A
Esrrb targeting gRNA Sequence1: 5’- ACCGAATGTCGTCCGAAGAC -3’ Sequence2: 5’- GCGAGTCCAGACCGTTGGCG -3’	This paper	N/A
Atf3 targeting gRNA Sequence1: 5’- GGCGGTCGCACTGACTTCTG -3’ Sequence2: 5’- CTTCCTTGACAAA GGGTGTC -3’	This paper	N/A
Puma targeting gRNA Sequence1: 5’- GCGCACGCCAGGAGGGCAGC -3’ Sequence2: 5’- CTCGGCCGCCTGATGCCCTC -3’	This paper	N/A
Noxa targeting gRNA Sequence1: 5’- GCAGCTCAACTCAGGAAGAT -3’ Sequence2: 5’- AGCCCAACCCGGGTGCCAGC -3’	This paper	N/A
shRNA-Esrrb targeting sequence 5’- GATTCGATGTACATTGAGA -3’	This paper	N/A
shRNA-Cdk9 targeting sequence 5’- GTACGAGAAACTTGCCAAGAT -3’	This paper	N/A
shRNA-Chek1 targeting sequence 5’- GCAACAGTTATTTCGGTATA -3’	This paper	N/A
shRNA-Chek2 targeting sequence 5’- CCTTCGTAAATACCGAGCTTA -3’	This paper	N/A

**Recombinant DNA**		

pET-21a	Novagen	Cat# 69740-3
PX459	Addgene	Cat# 48139
LentiCRISPR-V2	Addgene	Cat# 52961

**Software and algorithms**		

GraphPad Prism 8	GraphPad	N/A
FlowJo 10	FlowJo	N/A
ImageJ	ImageJ	N/A
Seahorse Wave Desktop	Agilent	N/A
DESeq2	v1.16.1	N/A
Hisat2	v2.0.5	N/A

**Other**		

TRIzol^TM^ Reagent	Invitrogen	Cat# 15596026
DMEM	Invitrogen	Cat# 11965092
Penicillin-Streptomycin	Invitrogen	Cat# 15140122
Puromycin	Invitrogen	Cat# A1113803
Fetal Bovine Serum	GEMINI	Cat# 900-108

### Resource availability

#### Lead contact

Further information and requests for resources and reagents should be directed to and will be fulfilled by the lead contact, Qintong Li (liqintong@scu.edu.cn).

#### Materials availability

All unique reagents generated in this study are available from the lead contact with a completed Material Transfer Agreement.

### Experimental model and subject details

#### Mice

Animal studies were approved by the Institutional Animal Care and Use Committee at West China Second University of Sichuan University (2020-028). Mice were housed under the Program of Laboratory Animal Center at West China Second University of Sichuan University with the principles and procedures of the Guide for the Care and Use of Laboratory Animals. All animals used in this study were female between 6 to 8 weeks. All mice had *ad libitum* access to food and water and were kept with 12-h light-dark cycles.

#### Cell lines

R1/E, E14Tg2a, F9 and HEK293T were purchased from ATCC, and verified to be free of mycoplasma by the PCR method. R1/E and E14Tg2a were routinely maintained as previously described (Dai et al., 2014). HEK293T, HCT116 and F9 cells were cultured in Dulbecco’s modified Eagle’s medium supplemented with 10% fetal bovine serum and penicillin-streptomycin. All cells were maintained at 37°C and 5% CO_2_. Most culture media were from Gibco unless otherwise noted. Heat-inactivated FBS was purchased from Gemini Bio-Products.

### Method details

#### Knockout cell line and cDNA

CRISPR/Cas9 genomic editing was used to generate knockout cell lines, with two gRNAs for each gene (Key resources table). Knockout cell lines were identified by protein blotting and DNA sequencing. To knock down gene expression, shRNA was used (Key resources table). Esrrb cDNA was cloned from R1/E total RNA (NCBI No. NM_001159500). Mutant Esrrb cDNA constructs were generated by Overlapping-Extension PCR (Hussain and Chong, 2016).

#### Recombinant proteins

Wildtype Esrrb and two mutant proteins, Esrrb 2A (S27A, S39A) and Esrrb 5A (S27A, S39A, S63A, S199A, S207A), were expressed as HIS-tagged proteins from a pET-21a vector in *E*. *coli* BL21 Star (DE3) cells. One-liter cells of each were induced with 1 mM IPTG overnight at 8°C once the OD_600_ reached 0.6. Cells were pelleted, resuspended in PBS, pelleted again, and then lysed in buffer containing 1X PBS, 0.1% TritonX-100, 5 mM imidazole, and 0.1% PMSF. Lysates were sonicated and subsequently salted up to 1 M NaCl prior to centrifugation for 45 min at 244,000 X *g* at 4°C. The resulting supernatants were each incubated with 1 mL of Ni-NTA agarose beads for 1 h with rotation at 4°C and washed with high salt buffer (10 mM Tris 7.8, 1 M NaCl, 35 mM imidazole, 1% PMSF, 0.1% TritonX-100), followed by low salt washes (10 mM HEPES 7.8, 50 mM KCl, 35 mM imidazole, 1% PMSF, 0.1% TritonX-100). Bound proteins were eluted with 10 mM HEPES 7.6, 50 mM KCl, 300 mM imidazole, 0.1% TritonX-100 and 1% PMSF, and were analyzed by 9% SDS-PAGE. Recombinant P-TEFb was generated as previously described (Li et al., 2005).

#### P-TEFb kinase assay

Recombinant wild-type and mutant Esrrb proteins were incubated with 0.04 pmol P-TEFb in 20 μL of buffer containing 20 mM HEPES 7.6, 50 mM K(Ac), 5 mM Mg(Ac), 50 ng/μL BSA, 30 μM ATP, 2.5 μCi γ-^32^P-ATP for 10 min at room temperature. Reactions were terminated with 5 μL of 5X PLB (20% Ficoll, 10% SDS, 50 mM Tris, 50 mM DTT) and analyzed using 9% SDS-PAGE with silver staining. The gel was dried and then visualized with a Fujifilm Typhoon FLA-7000 phosphor imager. The silver-stained gel was used to normalize the amounts of Esrrb loaded when quantifying the relative phosphorylation efficiency.

#### Quantitative PCR

Total RNA was extracted by Trizol (Invitrogen) following the manufacturer’s instructions, and reverse transcribed by random hexamers or oligo (dT) using M-MLV Reverse Transcriptase (Invitrogen). Quantitative PCR (qPCR) was carried out using Sosofast Eva Green Supermix (Bio-Rad, 1725201) on a Bio-Rad CFX96 Real-Time PCR Detection System. Primer sequences are listed in the Table S1. Expression levels of each gene were normalized to Gapdh or Tbp and quantified as previously described (Peirson et al., 2003).

#### Glycerol gradient analysis

Glycerol gradient analysis was performed as previously described (Li et al., 2007). Briefly, R1/E cells were lysed in buffer A (150 mM NaCl, 2 mM MgCl_2_, 10 mM HEPES, 1 mM EDTA, 1 mM DTT, 1% PMSF, EDTA-free protease inhibitor (Bimake, B14001), and 0.5% Nonidet P-40 for 15 min on ice. Cell lysates were centrifugation for 15 min at 13,000 rcf, and loaded onto 5 mL 5%–45% glycerol gradients in buffer A without Nonidet P-40. Gradients were run at 49,500 rpm for 16 h in a Beckman MLS-50 rotor, fractionated (300 μL each), and an aliquot from each fraction was analyzed by protein blotting.

#### Flow cytometry

Cells were stained with Annexin V-FITC and PI (BD Biosciences, 556547), 488 EdU Click Proliferation Kit (565455); MitoSOX red (Thermo Fisher Scientific, M36008), MitoTracker Green FM (M7514), and MitoTracker Red CM-H2XRos (M7513) following the manufacturer’s instructions. More than 10,000 events were collected by BD FACSCelesta, and analyzed by FlowJo V10 software.

#### Seahorse metabolic analysis

Real-time extracellular acidification rate (ECAR) was measured by Seahorse XFe24 Extracellular Flux Analyzer following the manufacturer’s instructions. Briefly, cells were seeded onto gelatin-coated 24-well plates at 1 × 10^5^ cells per well the day before measurement. The medium was changed to unbuffered DMEM (Sigma, D5030) supplemented with 2 mM L-glutamine and place the plate in a non-CO_2_ incubator equilibrated for 60 min at 37°C. ECAR was measured following the injection of: (i) glucose-free DMEM, (ii) 10 mM glucose, (iii) 10 μg/mL oligomycin (MCE, HY-N6782) and (iv) 50 mM 2-deoxyglucose (Sigma, D8375). After the last ECAR measurement, cells were counted using Countess 3 FL (Invitrogen), and cell number was used to normalize the ECAR value (mpH/min/10^4^ cells).

#### BH3 profiling assay

BH3 profiling assay was performed as previously described (Fraser et al., 2019). Briefly, R1/E cells were suspended in 25 μL DTEB buffer (135 mM trehalose, 50 mM KCl, 20 μM EDTA, 20 μM EGTA, 0.1% BSA, 5 mM succinate, 10 mM HEPES-KOH pH 7.5) at 2 × 10^6^ cells/mL, mixed with 25 μL of 4X Dig/Dye (4 mM JC-1, 40 μg/mL oligomycin, 20 mM 2-mercaptoethanol, 0.01% digitonin (w/v) digitonin in DTEB buffer), and stained for 10 min at room temperature. 50 μL of indicated peptide mimics dissolved in DTEB was then added to cells. Fluorescence at 590 nm was monitored using 545 nm excitation on a Varioskan multimode microplate reader (Thermo Fisher Scientific) at 30°C with automated readings every 15 min. The depolarization (%) was quantified as previously described (Ryan and Letai, 2013).

#### Teratoma assay

Animal studies were approved by the Institutional Animal Care and Use Committee at West China Second University of Sichuan University (2020-028). Mice were housed under the Program of Laboratory Animal Center at West China Second University of Sichuan University with the principles and procedures of the Guide for the Care and Use of Laboratory Animals. Teratoma formation was carried out as previously described (Nelakanti et al., 2015). Briefly, WT, EKO, EKO + WT, EKO+5A cells were resuspended in PBS at 5 × 10^6^ cell/mL. 100 μL of cells (5 × 10^5^ cells) were injected subcutaneously into flanks of NOG-dKO female mice (Yaguchi et al., 2018). For drug treatment, PBS or cisplatin (3.5 mg/kg) was injected intraperitoneally. Tumor size was constantly monitored, and mice were sacrificed when tumor volume reached to approximately 1 cm^3^. In this study, because Esrrb+5A cells grew the fastest and reached to 1 cm^3^ limitation first, mice inoculated with WT, EKO, EKO + WT were also sacrificed at the same time point. To measure the efficacy *in vivo*, tumor growth inhibition ratio (TGI) was used, defined using the following formula: TGI (%) = [1 − (weight of the treated group)/(weight of the control group)] X 100 (%) (Pan et al., 2020).

#### RNA sequencing and data analysis

For each experiment, three independent biological replicas were subjected to transcriptomic analysis by RNA sequencing (RNA-seq). Sequencing libraries were generated using NEBNext® UltraTM RNA Library Prep Kit for Illumina® (NEB, USA), and index codes were added to track the identity of each sample. The library preparations were sequenced on an Illumina Novaseq platform to generate 150 bp paired-end reads. The resulting fastq files were quality controlled using FastQC v0.11.8, and adapters were trimmed using Trimmomatic v0.39. Paired-end clean reads were aligned to the reference genome (GRCh38.p12) using Hisat2 v2.0.5. featureCounts v1.5.0-p3 was used to count the reads numbers mapped to each gene. Transcripts per kilobase of exon model per million mapped reads (TPM) was calculated based on the length of the gene and reads count mapped to this gene. Differential gene expression analysis was performed using the DESeq2 R package (1.16.1). Gene Set Enrichment Analysis (GSEA) was performed using the lists of hallmark gene sets from the Molecular Signature Database (MsigDB) using default parameters (Liberzon et al., 2015). Transcription factor enrichment analysis using ChEA3 was performed as previously described (Keenan et al., 2019). Heatmap was generated using the heatmap package from R.

### Quantification and statistical analysis

Representative results from at least three independent experiments were shown. Statistical comparisons were made by using Student’s *t* test with GraphPad Prism 8. The significance of difference was set at p values <0.05. In all figures, ∗ denotes p < 0.05, ∗∗ denotes p < 0.01, ∗∗∗ denotes p < 0.001, ∗∗∗∗ denotes p < 0.0001.

## Data Availability

• RNA-seq data have been deposited at SRA database under accession number: PRJNA827641. All data reported in this paper will be shared by the lead contact upon request. • This study did not generate original code. • Any additional information required to reanalyze the data reported in this paper is available from the lead contact upon request.
