# Supplementary material for: Dynamic regulation of P-TEFb by 7SK snRNP is integral to the DNA damage response to regulate chemotherapy sensitivity
Source: iScience. 2022 Aug 4;25(9):104844. doi: 10.1016/j.isci.2022.104844 (PMC9399290; doi:10.1016/j.isci.2022.104844)

iScience, Volume *25*

Supplemental information

Dynamic regulation of P-TEFb by 7SK snRNP is integral to the DNA damage response to regulate chemotherapy sensitivity

Yin Fang, Yan Wang, Benjamin M. Spector, Xue Xiao, Chao Yang, Ping Li, Yuan Yuan, Ping Ding, Zhi-Xiong Xiao, Peixuan Zhang, Tong Qiu, Xiaofeng Zhu, David H. Price, and Qintong Li

SUPPLEMENTAL INFORMATION

Figure S1. Proteasomal degradation of Esrrb protein is independent of canonical DNA damage pathways, Related to Figure 2

(A) The effect of chemical inhibitors of ATM (KU-60019, 5 μM), ATR (VE821, 5 μM) and DNA-PK (NU7441, 2μM) on cisplatin (Cis, 5 μM)-induced Esrrb degradation. Increased Cdc25a protein level is a well-established marker of the inhibition of the ATR/ATM/DNA-PK-CHK1/2 axis.

(B) The combinatorial effect of chemical inhibitors of ATM, ATR and DNA-PK on cisplatin (Cis, 5 μM)-induced Esrrb degradation.

(C) The effect of chemical inhibitor of CHK1/2 (AZD7762, 10 μM) on cisplatin (Cis, 5 μM)-induced Esrrb degradation.

(D) The effect of chemical inhibitor of p38 (SB203508, 25 μM) on cisplatin (Cis, 5 μM)-induced Esrrb degradation.

(E) The effect of chemical inhibitor of CHK1/2, or together with Chk1 knockdown, on UV (UV, 10 J/m^2)-induced Esrrb degradation.

(F) The effect of knockdown of Chk1 and Chk2 individually, or in combination, on UV (UV, 10 J/m^2)-induced Esrrb degradation.

(G) qPCR analysis of the efficiency of Chk2 knockdown by shRNA.

Figure S2. Proteasomal degradation of Esrrb protein is not regulated by p53 or Nanog, Related to Figure 2

(A) The effect of proteasome inhibitor MG132 (5 μM) on cisplatin -induced Nanog protein degradation (Cis, 5 μM).

(B) The effect of proteasome inhibitor MG132 (5 μM) on UV-induced Nanog protein degradation (UV, 10 J/m^2).

(C) The effect of proteasome inhibitor MG132 (5 μM) on doxorubicin -induced Nanog protein degradation (Dox, 1 μM).

(D) The effect of UV (UV, 10 J/m^2) on Nanog mRNA level in wild-type (WT) and p53 knockout (KO) cells (left panel), and the effect of cisplatin (Cis, 5 μM) on Nanog mRNA level (right panel) (n=3). The bar plot represents mean ± SD. **, $P < 0.01$; ****, $P < 0.0001$ (unpaired t test).

(E) The effect of UV (UV, 10 J/m^2) on Nanog and Esrrb protein levels in WT and p53 KO cells.

(F) The effect of P-TEFb inhibitor NVP-2 on Nanog mRNA level. The bar plot represents mean ± SD. ***, $P < 0.001$ (unpaired t test).

Figure S3. Chemical inhibition of CDK1/2/4/6/7/12/13 does not rescue cisplatin-induced Esrrb proteasomal degradation, Related to Figure 2

(A-D) The effect of chemical inhibition of CDK1/2 (RO-3306, 3 μM) **(A)**, CDK4/6 (Palbociclib, 1μM) **(B)**, CDK7 (THZ1, 250 nM) **(C)** and CDK12/13 (THZ531, 1 μM) **(D)** on cisplatin-induced Esrrb proteasomal degradation.

Figure S4. P-TEFb inhibitor rescues UV-induced Esrrb degradation, Related to

Figure 2

The effect of P-TEFb chemical inhibitor DRB at indicated concentration on UV - induced Esrrb proteasomal degradation (UV, 10 J/m^2).

Figure S5. The pluripotent cellular state of WT, EKO, EKO+WT and EKO+5A cells are indistinguishable, Related to Figure 4

(A) Western blot analysis of the expression of endogenous as well as reconstituted Esrrb, and Oct4 in WT, EKO, EKO+WT and EKO+5A cells. Oct4 is a master regulator of pluripotency.

(B) Cell cycle profiling by FACS analysis of WT, EKO, EKO+WT and EKO+5A cells. Edu, 5-ethynyl-2'-deoxyuridine. PI, propidium iodide.

(C) FACS analysis of Pecam-1 expression in WT, EKO, EKO+WT and EKO+5A cells. Pecam-1 is a widely used cell surface marker of pluripotency. Iso, antibody isotype control.

(D) Alkaline phosphatase (AP) staining analysis of WT, EKO, EKO+WT and EKO+5A cells. Positive AP staining is a well-established marker of pluripotency.

(E) The expression level of lineage-specific transcripts in WT, EKO, EKO+WT and EKO+5A cells by RNA-seq analysis. Note that the expression level of all transcripts is low (TPM < 1).

(F) Heatmap presentation of the expression of pluripotent genes (KEGG pathway: mmu04550) analyzed by RNA-seq in WT, EKO, EKO+WT and EKO+5A cells (n=3). Colored scale bar denotes z-score value.

(G) Rescue of potential Esrrb target genes (756 genes) by reconstituted WT or 5A mutant Esrrb via RNA-seq analysis (n=3). 622 genes (82.3%) and 616 genes (81.5%) were rescued by WT and 5A mutant Esrrb, respectively.

Figure S6. Reanalysis of published Esrrb ChIP-seq datasets, Related to Figure 4

(A) Venn diagram presentation of the number of Esrrb-regulated genes inferred from published ChIP-seq studies. A gene is regarded as a potential Esrrb-regulated gene if Esrrb was found to bind within 2,000 bp of its transcriptional start site by ChIP-seq.

(B) Venn diagram presentation of potential Esrrb target genes identified in **(A)** and differentially expressed genes in EKO identified by this study.

(C) Heatmap presentation of the expression of 97 potential Esrrb target genes in **(B)** analyzed by RNA-seq (n=3). Colored scale bar denotes z-score value.

(D) qPCR analysis of potential Esrrb-regulated genes in Esrrb knockout (EKO) or knockdown (Esrrb-shRNA) cells (n=3).

Figure S7. Multiple mitochondrial parameters are indistinguishable in WT, EKO, EKO+WT and EKO+5A cells, Related to Figure 4

(A) Mitochondrial priming, that is, the apoptotic threshold, is highly similar in WT, EKO, EKO+WT and EKO+5A cells, determined by BH3 profiling assay (n=3). PUMA2A is a negative control. BIM and BID peptides are inducers of mitochondrion-mediated cell death.

(B) FACS analysis of mitochondrial content, membrane integrity and ROS in WT, EKO, EKO+WT and EKO+5A cells.

Figure S8. Chemical inhibition of P-TEFb largely abolishes UV-induced cell death, Related to Figure 5

FACS analysis of cell viability (left panel) induced by UV (UV, 10 J/m^2), in the absence or presence of P-TEFb chemical inhibitor flavopiridol (Flavo, 200 nM). Cells negative for both annexin V and propidium iodide (PI) were defined as live cells. Numbers denote the percentage of cells in each population, with that of live cells labeled in red. Right panel, the quantification of the percentage of viable cells by FACS analysis (n=3). The bar plot represents mean ± SD. ***, $P < 0.001$ (unpaired t test).

Figure S9. Atf3 is P-TEFb dependent, p53 target gene induced by DNA damage, but does not contribute to cisplatin-induced cell death, Related to Figure 6

(A and B) The effect of cisplatin (Cis, 5 μM) and P-TEFb inhibitor NVP-2 (20 nM) on Atf3 mRNA (n=3) **(A)** and protein level **(B)**. The bar plot represents mean ± SD. ****, $P < 0.0001$ (unpaired t test).

(C and D) The effect of UV (10 J/m^2) and P-TEFb inhibitor flavopiridol on Atf3 mRNA (n=3) **(C)** and protein level **(D)**. The bar plot represents mean ± SD. *, $P < 0.05$; ***, $P < 0.001$ (unpaired t test).

(E) The effect of UV (10 J/m^2) and P-TEFb inhibitor NVP-2 on Atf3 and Esrrb protein expression.

(F) Generation of Atf3 knockout cells by CRISPR/Cas9. Cisplatin (Cis, 5 μM) was unable to induce detectable Atf3 protein expression in knockout cells.

(G) The effect of Atf3 knockout on cisplatin -induced cell death (Cis, 5 μM) (n=3).

(H) Generation of p53 knockout cells by CRISPR/Cas9. Cisplatin (Cis, 5 μM) and MG132 (5 μM) treatments were unable to induce detectable p53 protein expression in knockout cells.

(I and J) The effect of p53 on UV-induced (10 J/m^2) Atf3 mRNA **(I)** and protein expression **(J)**. The bar plot represents mean ± SD. *, $P < 0.05$ (unpaired t test).

Figure S10. P-TEFb regulates UV-induced transcriptional program, Related to Figure 6

(A) Pie chart presentation of the P-TEFb dependency of UV-regulated, early-response genes by RNA-seq (6 hour after UV treatment). The percentage inside the pie chart denotes the percentage of genes unaltered (73.74%), downregulated (15.48%) or upregulated (10.78%) by UV. The percentage outside the pie chart denotes the percentage of genes upregulated by UV in a P-TEFb dependent manner (35.79%). Of note, suboptimal concentration of NVP-2 (10 nM), indicated by partial inhibition of RNA pol II CTD Ser2 phosphorylation, was used in this experiment to detect genes most sensitive to P-TEFb inhibition.

(B) Transcription factor enrichment analysis of UV-induced, P-TEFb dependent

genes by ChIP-X Enrichment Analysis 3 (ChEA3). Top ten transcription factors predicted are listed and ranked by false discovery rate (FDR). Vertical dashed line denotes FDR = 0.01. Out of 982 gene promoters bound by p53 in ChEA3, 539 were expressed in UV-treated R1/E cells (TPM > 1).

(C) Gene set enrichment analysis (GSEA) of the effect of P-TEFb inhibitor NVP-2 on UV-induced pathways. p53 transcriptional program was shown as an example. NES, normalized enrichment score. FDR, false discovery rate.

(D) Pie chart presentation of the dependency of UV-regulated genes on P-TEFb and p53.

Figure S11. Pearson correlation analysis of all RNA-seq experiments in this study, Related to Figures 4, 6, S5, 6 and 10

For each group, RNA-seq was carried out for three independent biological replicates. Pearson correlation is > 0.965 for cisplatin-treated groups **(A)**, > 0.939 for UV-treated groups **(B)**, and > 0.982 for untreated group **(C)**. Note that RNA-seq was carried out independently for wild-type cells in each treatment group. Data from one sample of wild-type cells in group **(C)** did not pass the quality control for sequencing, and was excluded from downstream analysis.

Figure S12. Working model of P-TEFb regulating DNA-damage response to determine cellular fate, Related to Figures 1-6 and S1-S10

Acute DNA damage signals, such as cisplatin, UV and doxorubicin, activate P-TEFb

by releasing it from inhibitory 7SK snRNP. Activated P-TEFb can directly phosphorylate transcription factor ESRRB to promote its proteasomal degradation and cell survival, but also is required for the transcription of numerous p53 target genes to induce cell death. A recent study suggested that ATF3 functions as a transcriptional repressor after UV irradiation (Epanchintsev et al., 2017). However, knockout of ATF3 did not affect DNA damage-induced cell death in the present study. Thus, the function of P-TEFb regulated transcription factors, such as ATF3, remain to be clarified. Ultimately, the combinatorial effects of these transcription factors determine the cell fate in the response to the DNA damage.

Table S1. qPCR primers, Related to STAR Methods.

Data S1. Full images of western blots, Related to Figures 1-6 and S1-S10

Full unprocessed images of western blot, with the regions used in the corresponding Figures indicated by red dashed frames.

Figure S1. Proteasomal degradation of Esrrb protein is independent of canonical DNA damage pathways, Related to Figure 2.

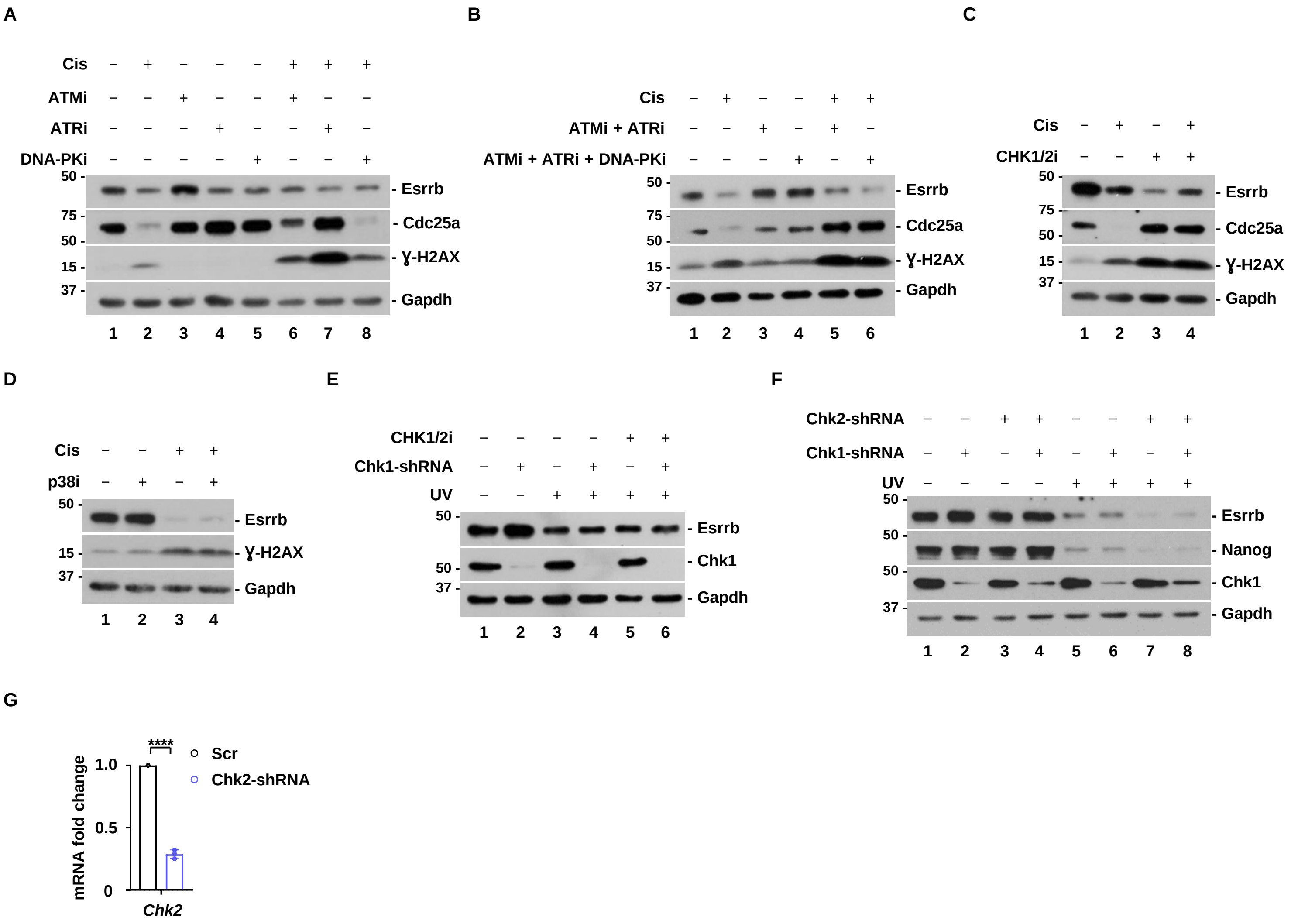

Figure S2. Proteasomal degradation of Esrrb protein is not regulated by p53 or Nanog, Related to Figure 2.

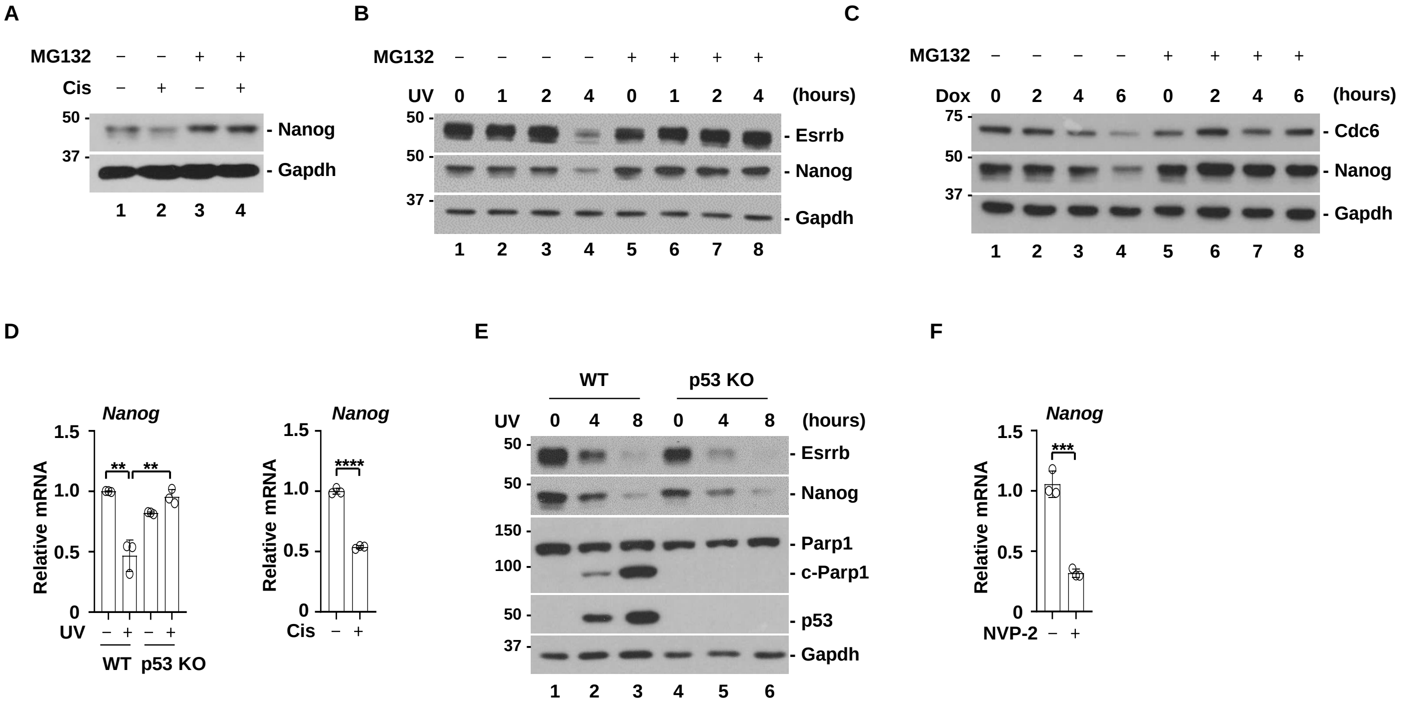

Figure S3. Chemical inhibition of CDK1/2/4/6/7/12/13 does not rescue cisplatin-induced Esrrb proteasomal degradation, Related to Figure 2.

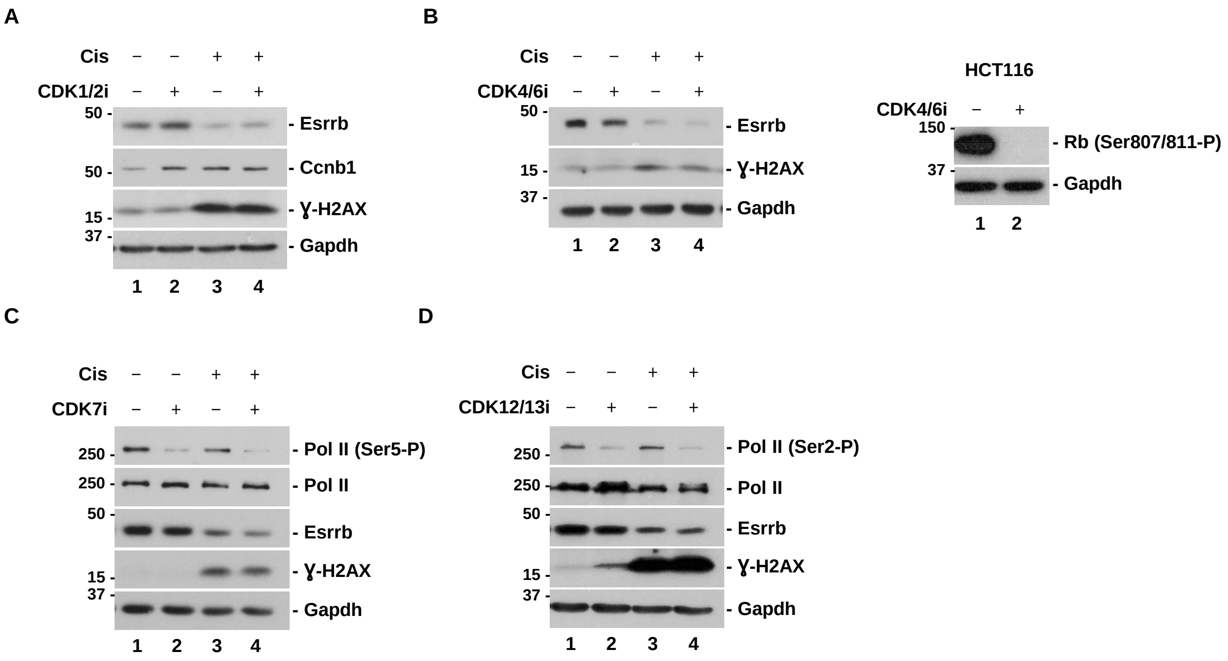

Figure S4. P-TEFb inhibitor rescues UV-induced Esrrb degradation, Related to Figure 2.

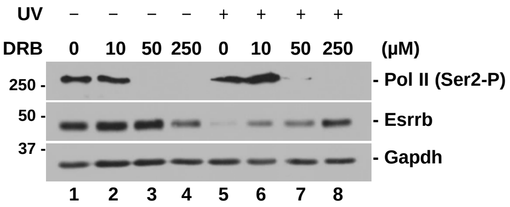

Figure S5. The pluripotent cellular state of WT, EKO, EKO+WT and EKO+5A cells are indistinguishable, Related to Figure 4.

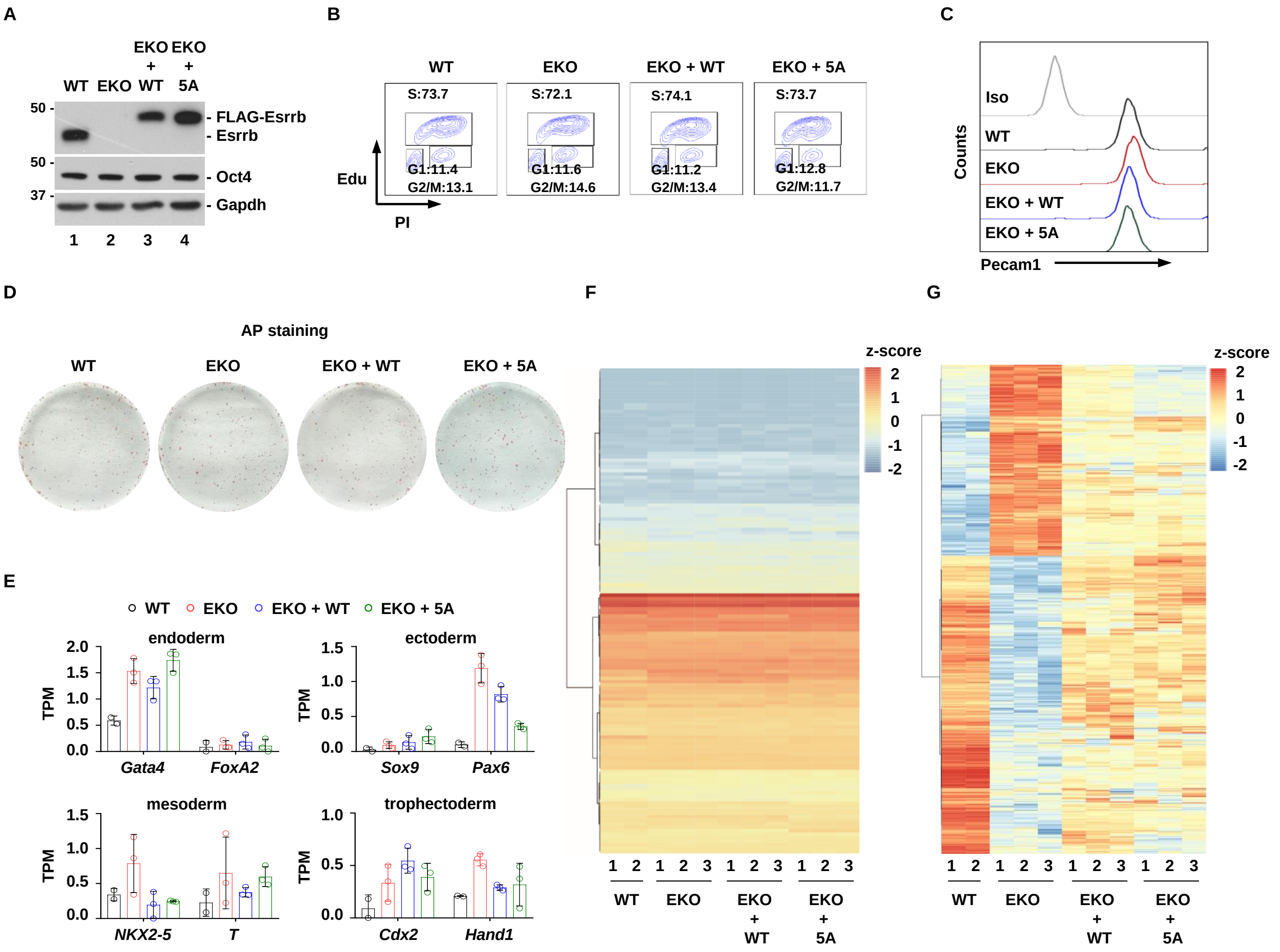

Figure S6. Reanalysis of published Esrrb ChIP-seq datasets, Related to Figure 4.

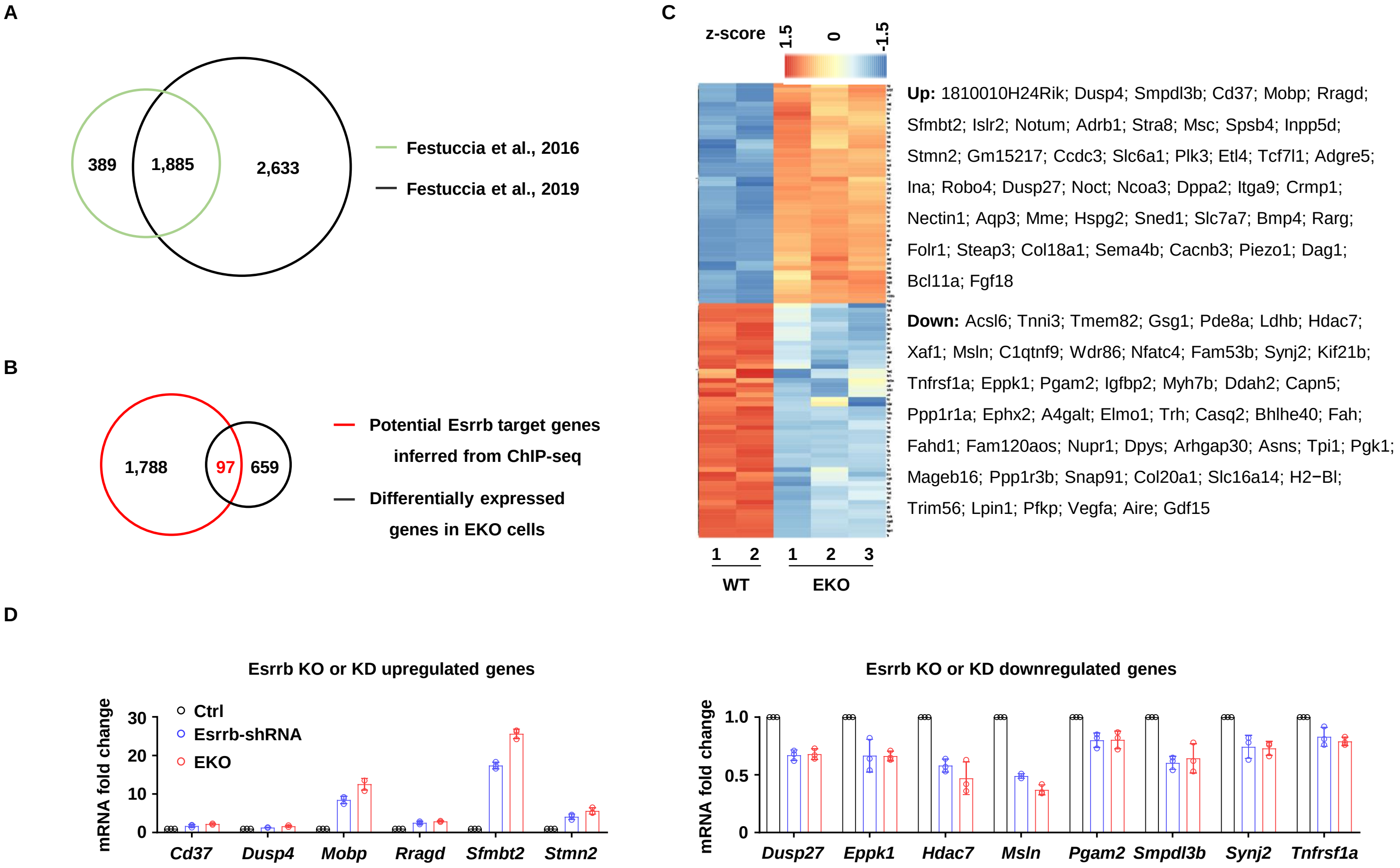

Figure S7. Multiple mitochondrial parameters are indistinguishable in WT, EKO, EKO+WT and EKO+5A cells, Related to Figure 4.

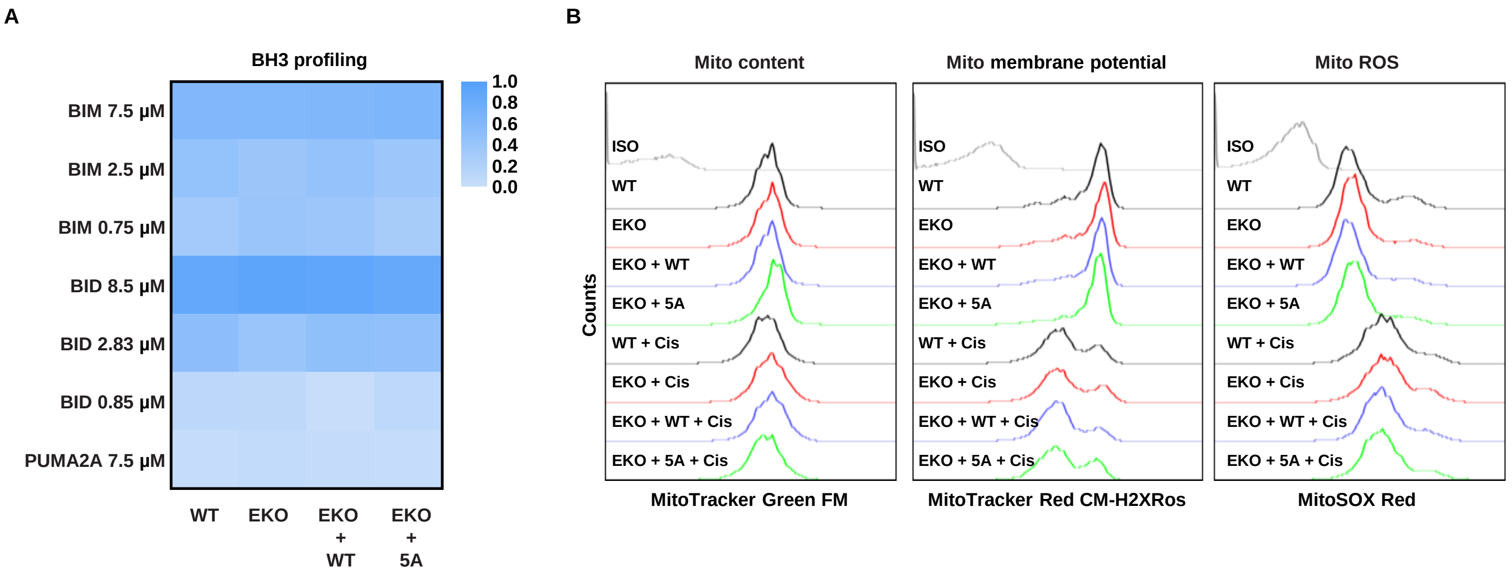

Figure S8. Chemical inhibition of P-TEFb largely abolishes UV-induced cell death, Related to Figure 5.

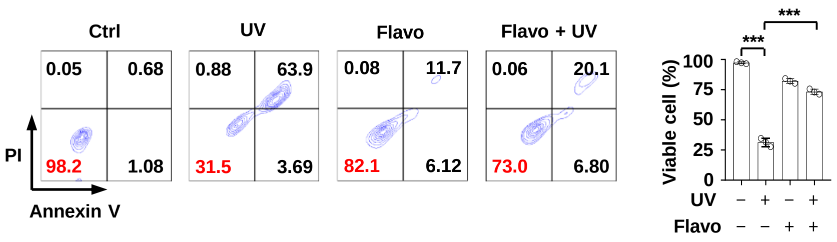

Figure S9. Atf3 is P-TEFb dependent, p53 target gene induced by DNA damage, but does not contribute to cisplatin-induced cell death, Related to Figure 6.

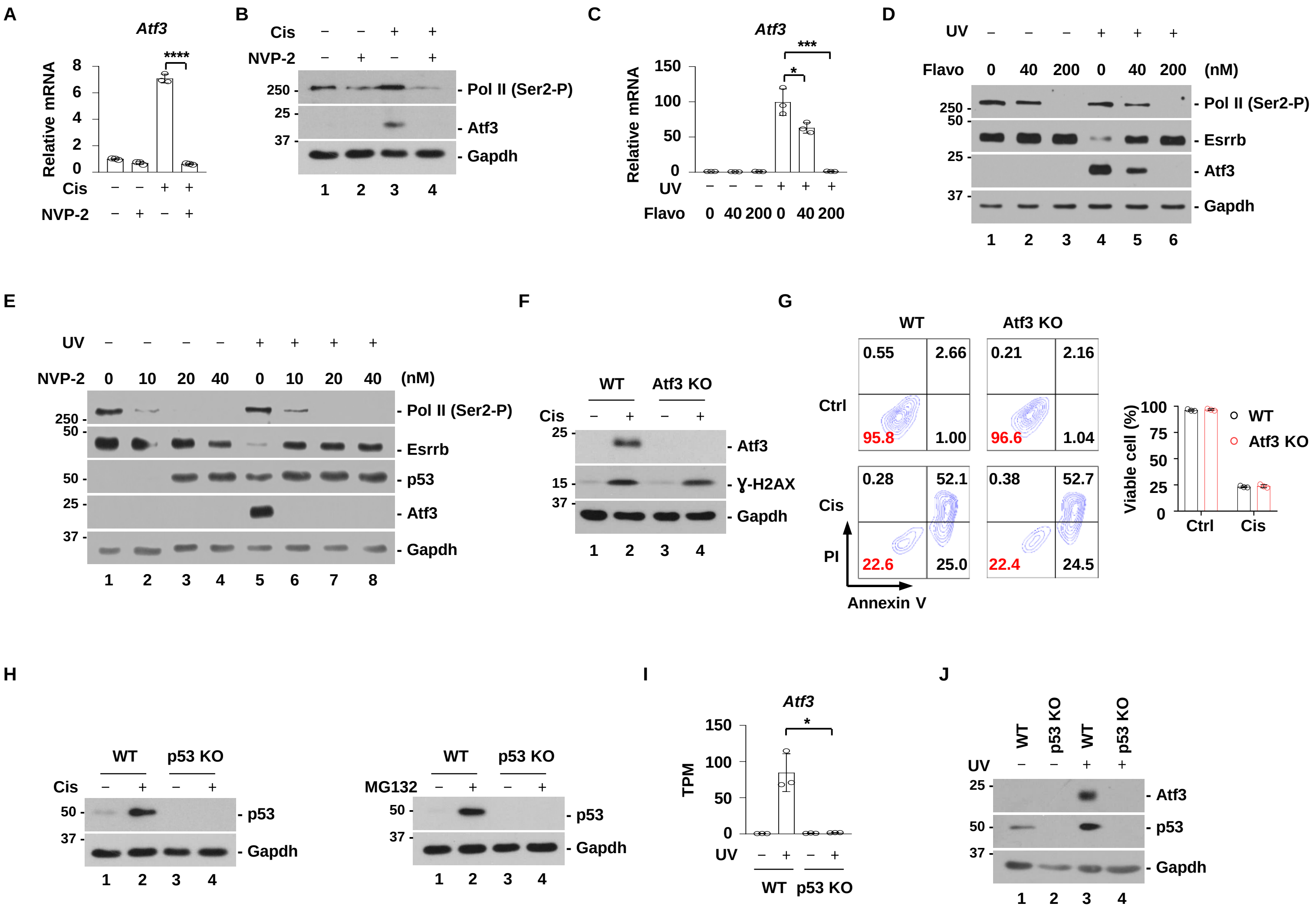

Figure S10. P-TEFb regulates UV-induced transcriptional program, Related to Figure 6.

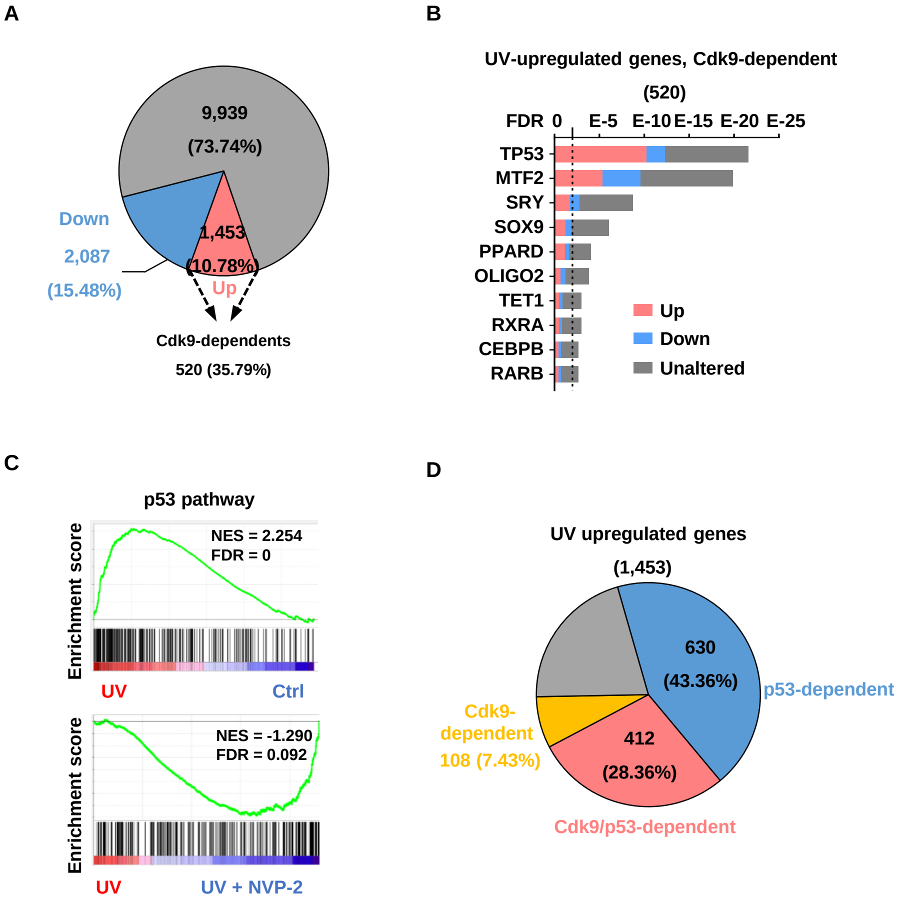

Figure S11. Pearson correlation analysis of all RNA-seq experiments in this study, Related to Figures 4, 6, S5, 6 and 10.

A

WT

	1	2	3
1	1.000	0.999	0.999
2	0.999	1.000	0.998
3	0.999	0.998	1.000

WT + Cis

	1	2	3
1	1.000	0.999	0.999
2	0.999	1.000	0.999
3	0.999	0.999	1.000

WT + NVP-2

	1	2	3
1	1.000	0.998	0.996
2	0.998	1.000	0.999
3	0.996	0.999	1.000

WT + Cis + NVP-2

	1	2	3
1	1.000	0.965	0.999
2	0.965	1.000	0.968
3	0.999	0.968	1.000

p53 KO

	1	2	3
1	1.000	0.999	0.998
2	0.999	1.000	0.999
3	0.998	0.999	1.000

p53 KO + Cis

	1	2	3
1	1.000	0.999	0.999
2	0.999	1.000	0.999
3	0.999	0.999	1.000

R^2: 1.00, 0.95, 0.90

B

WT

	1	2	3
1	1.000	0.997	0.997
2	0.997	1.000	1.000
3	0.997	1.000	1.000

WT + UV

	1	2	3
1	1.000	0.994	0.994
2	0.994	1.000	0.996
3	0.994	0.996	1.000

WT + NVP-2

	1	2	3
1	1.000	0.997	0.955
2	0.997	1.000	0.939
3	0.955	0.939	1.000

WT + UV + NVP-2

	1	2	3
1	1.000	0.996	0.995
2	0.996	1.000	0.999
3	0.995	0.999	1.000

p53 KO

	1	2	3
1	1.000	0.998	0.998
2	0.998	1.000	1.000
3	0.998	1.000	1.000

p53 KO + UV

	1	2	3
1	1.000	0.990	0.989
2	0.990	1.000	1.000
3	0.989	1.000	1.000

R^2: 1.00, 0.95, 0.90

C

WT

	1	2
1	1.000	0.981
2	0.981	1.000

EKO

	1	2	3
1	1.000	0.985	0.980
2	0.985	1.000	0.982
3	0.980	0.982	1.000

EKO + WT

	1	2	3
1	1.000	0.986	0.985
2	0.986	1.000	0.985
3	0.985	0.985	1.000

EKO + 5A

	1	2	3
1	1.000	0.983	0.986
2	0.983	1.000	0.983
3	0.986	0.983	1.000

R^2: 1.00, 0.95, 0.90

Figure S12. Working model of P-TEFb regulating DNA-damage response to determine cellular fate, Related to Figures 1-6 and S1-S10.

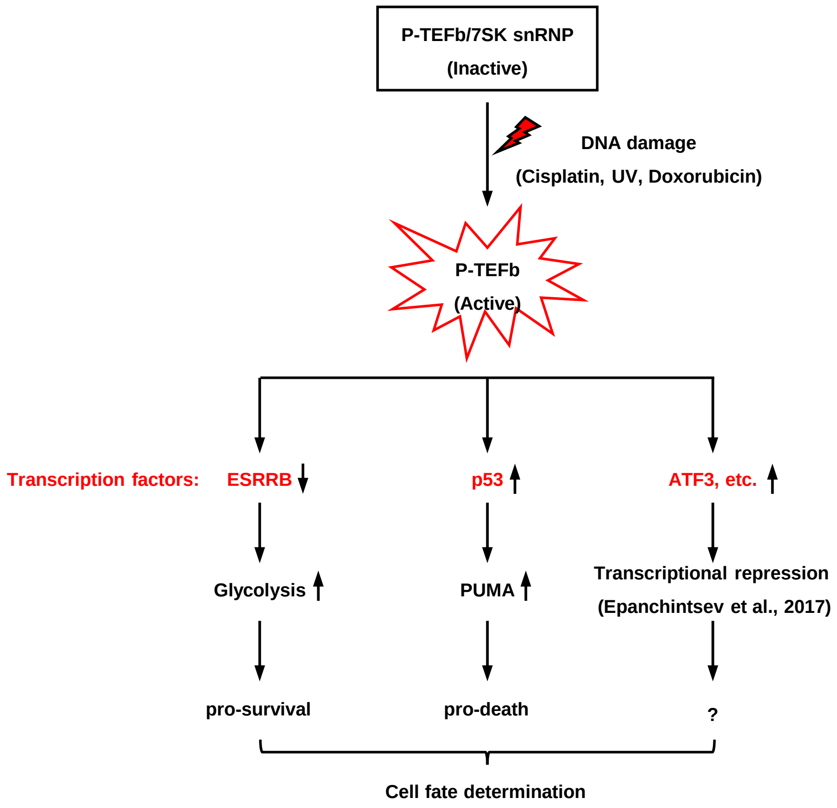

Table S1.   qPCR primers,   Related to STAR METHODS.
Esrrb **:**<br>Forward: AGCCAAGCAACGAGTCATG<br>Reverse: GATGAAGGAGCCGCAACTAG
Ldhb **:**<br>Forward: ACAAGTGGGTATGGCATGTG<br>Reverse: ACAATTTTCGGAGTCTGGAGG
Xaf1 **:**<br>Forward: CTCTCCACTTCATGCTCCAC<br>Reverse: GTGCTGTTGGCTTTCCTTG
Atf3 **:**<br>Forward: ATAAACACCTCTGCCATCGG<br>Reverse: GCCTCCTTTTCCTCTCATCTTC
Nanog **:**<br>Forward: AGCAGATGCAAGAACTCTCCTCCA<br>Reverse: CCGCTTGCACTTCATCCTTTGGTT
Cd37 **:**<br>Forward: TGCAAAAGCCCACATCTACC<br>Reverse: ACACAGGAATATTGAGAGCGTC
Dusp4 **:**<br>Forward: GTACCTCCCAGCACCAATG<br>Reverse: ATCCAACATGTCCCTGCG
Mobp **:**<br>Forward: CAAATGAGAGCAAGACAAGCG<br>Reverse: AACTTCTGGTTCTTGGAGAGC
Rragd **:**<br>Forward: TTTGACCCTACCTTTGACTATGAG<br>Reverse: ATCAGTGTTCACCTTGTAGGC
Sfmbt2 **:**<br>Forward: GGGAATGGGACTCTTTACTCAG<br>Reverse: GTTACTCCGAATGCTGATCTCC
Stmn2 **:**<br>Forward: CACATCCCTACAATGGCTAAAAC<br>Reverse: TTGATCTGCTTCACCTCCATG
Dusp27 **:**<br>Forward: AGCTGAGTTCCTTGACGAAG<br>Reverse: TGCTGATACCCATTTCACTGC
Eppk1 **:**<br>Forward: GACTTGGGTCAACATAGGGAG<br>Reverse: GTGGCACAGAACTTGCTTAAG
Hdac7 **:**<br>Forward: CTGTCCAGAACCCAGTCTTC<br>Reverse: TGTTTCAGCATCACCGAGTC

Msln **:**<br>Forward: AACGAGATTCCCTTCACCTATG<br>Reverse: GTGGATGTCTTCAGGGCTAAC
Pgam2 **:**<br>Forward: CGCACCTAAGATTAAGGCTGG<br>Reverse: TCCAGCTCATACACAATGGG
Smpdl3b **:**<br>Forward: CATGACTTCCACCCTAAGAACC<br>Reverse: CCCGGCAACTTCTCAGAATAG
Synj2 **:**<br>Forward: AACTCAACCTTCTAGACAGCG<br>Reverse: GAACCTCCACCTCCACAATG
Tnfrsf1a :<br>Forward: CTCTGCTCTACGAATCACTCTG<br>Reverse: CACAGCATACAGAATCGCAAG
Chk2:<br>Forward: CTGAGGACCAAGAACCTGAAG<br>Reverse: CCATCGAAGCAATATTCACAGC
Gapdh **:**<br>Forward: TGCACCACCAACTGCTTAGC<br>Reverse: GGCATGGACTGTGGTCATGAG
Tbp **:**<br>Forward: GGGGAGCTGTGATGTGAAGT<br>Reverse: CCAGGAAATAATTCTGGCTCA

Data S1. Full images of western blots, Related to Figures 1-6 and S1-S10.

Figure 1A

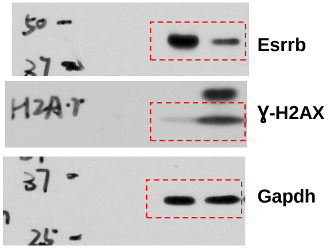

Figure 1B

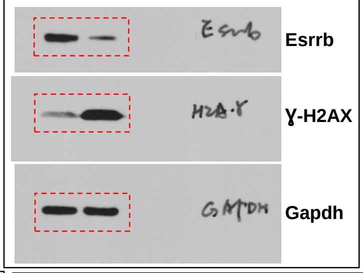

Figure 1C

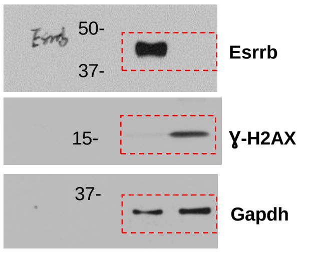

Figure 1E

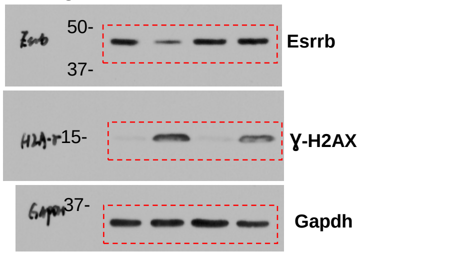

Figure 1F

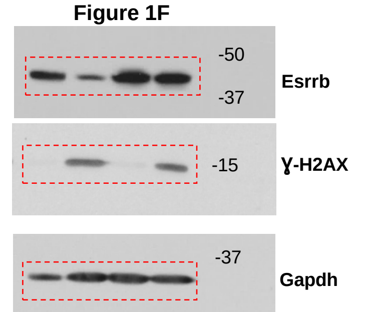

Figure 1G

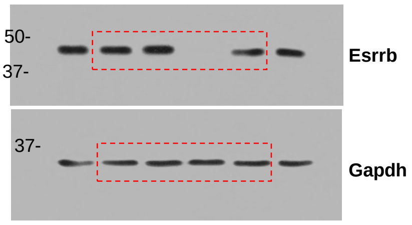

Figure 1H

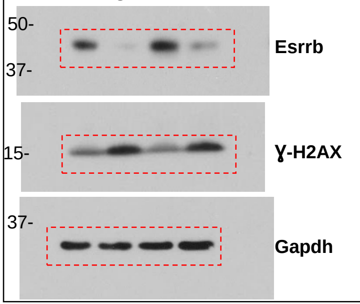

Figure 1J

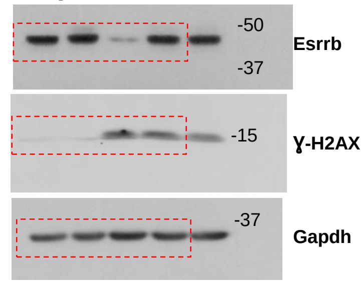

Figure 1I

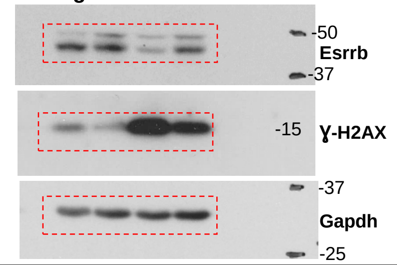

Figure 2A

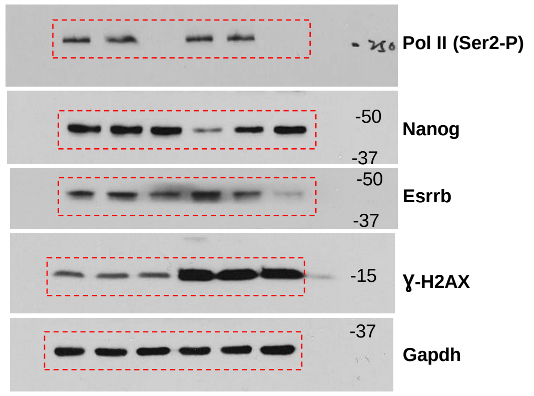

Figure 2B

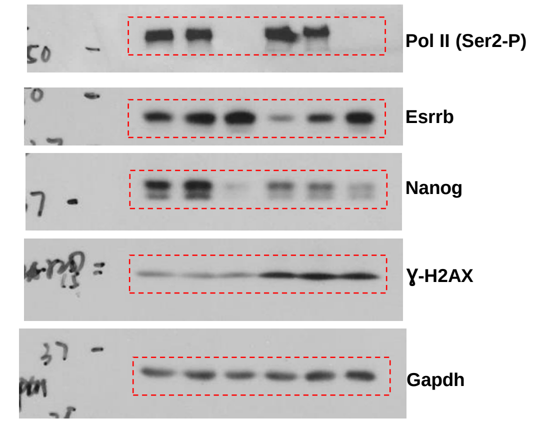

Figure 2C

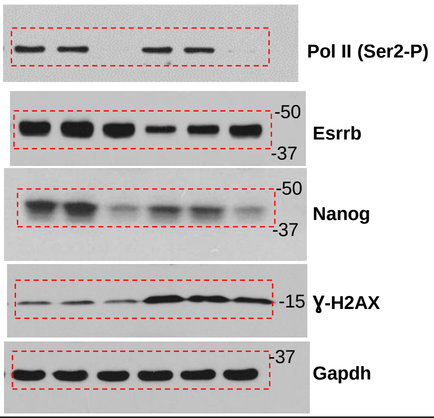

Figure 2E

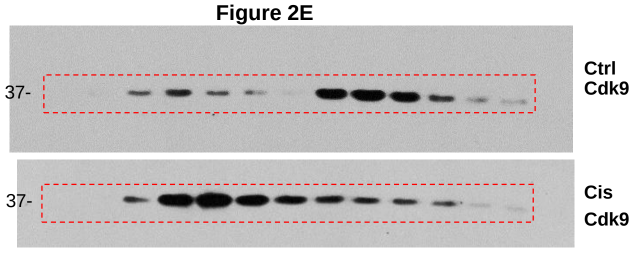

Figure 2G

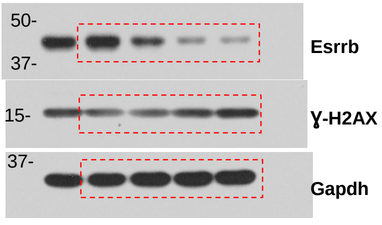

Figure 2I

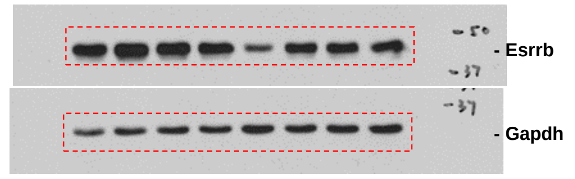

Fig.2F

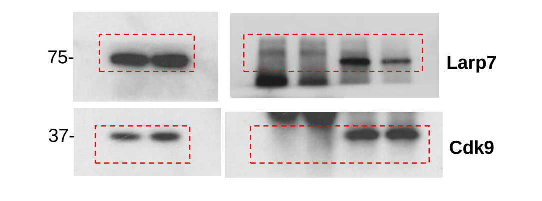

Fig.2H

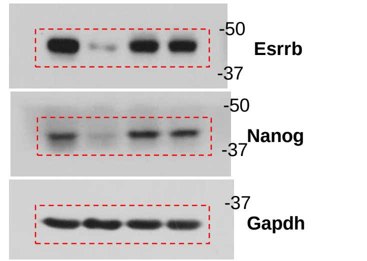

Figure 2J

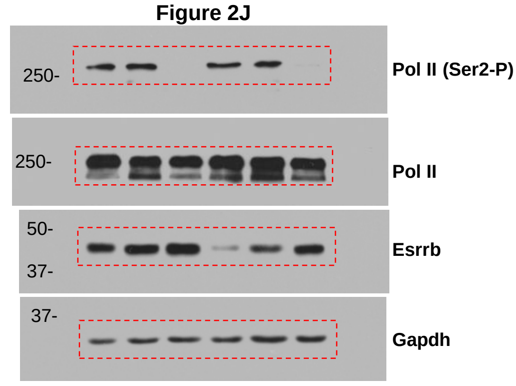

Figure 2K

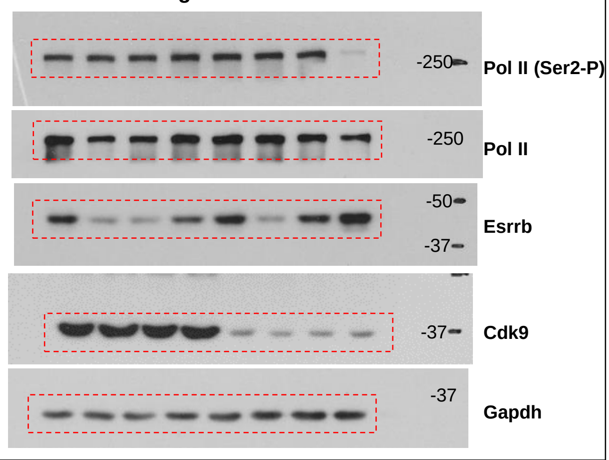

Figure 2L

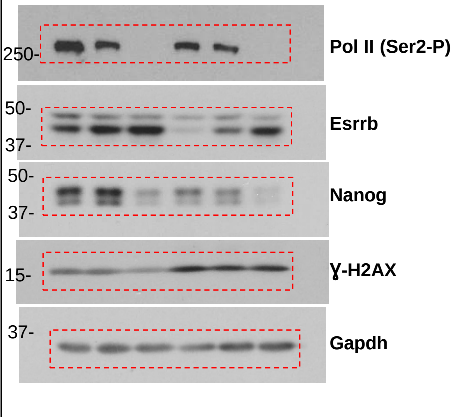

Figure 2M

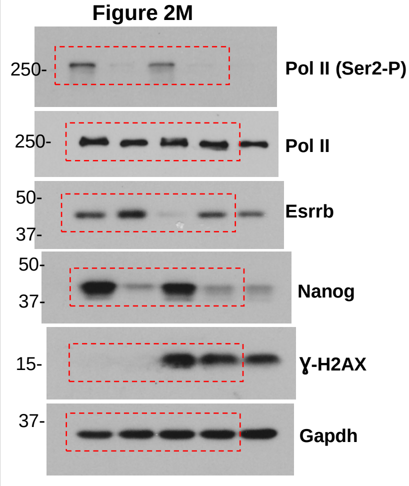

Figure 2N

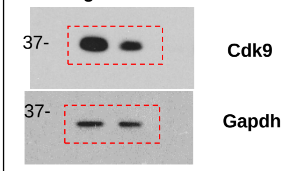

Figure 3C

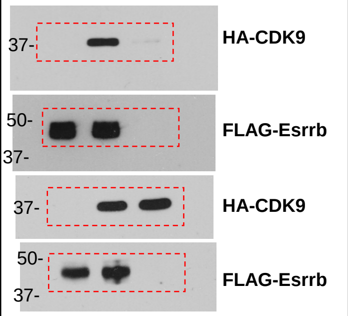

Figure 2O

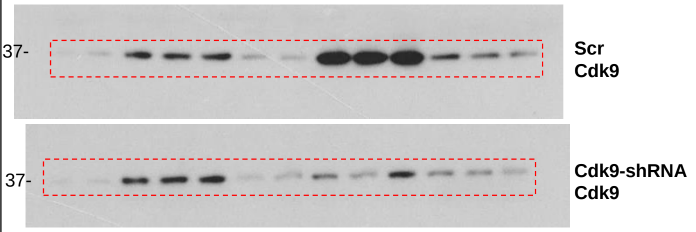

Figure 3D

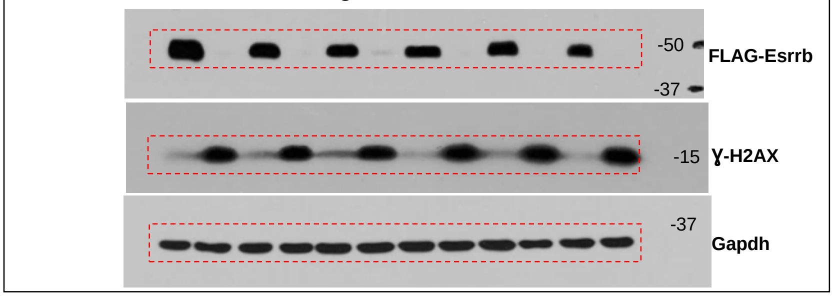

Figure 3E

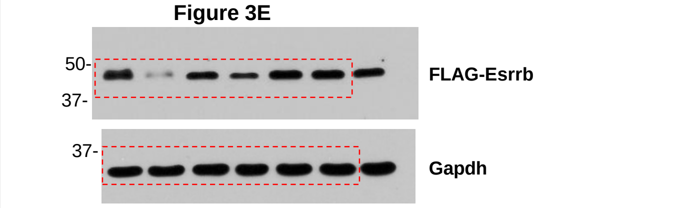

Figure 3F

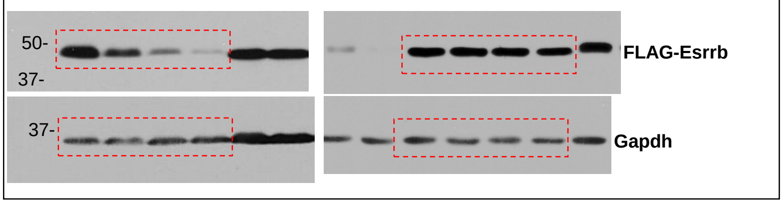

Figure 3I

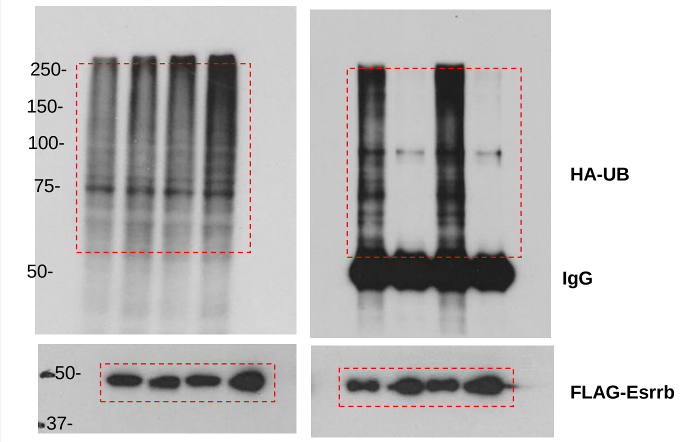

Figure 3H

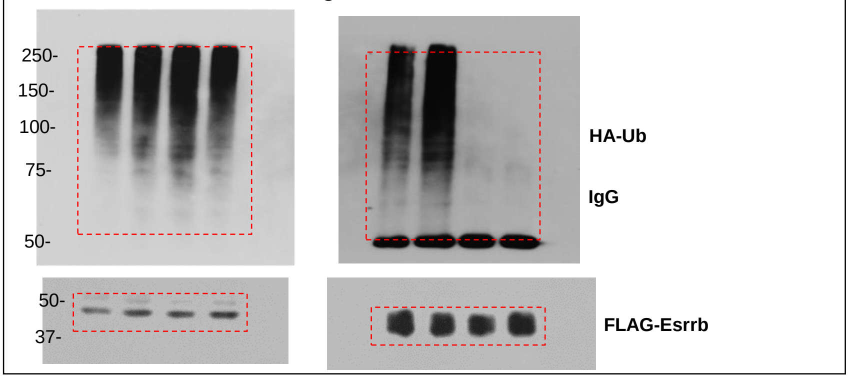

Figure 4C

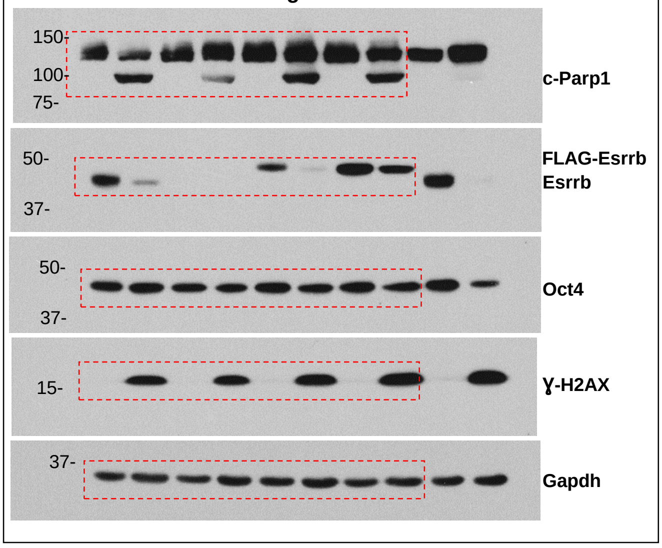

Figure 4G

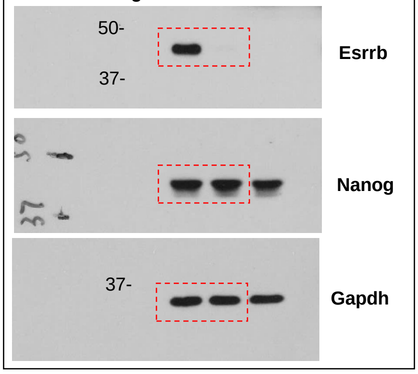

Figure 6I

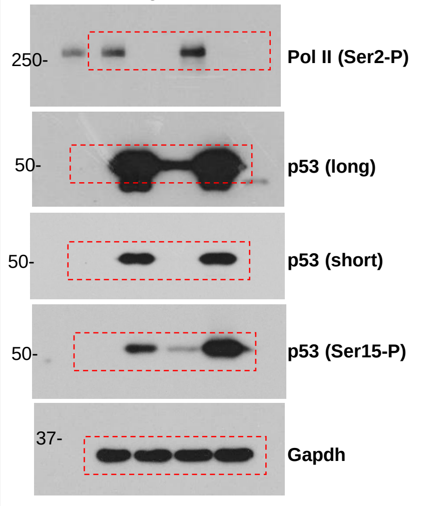

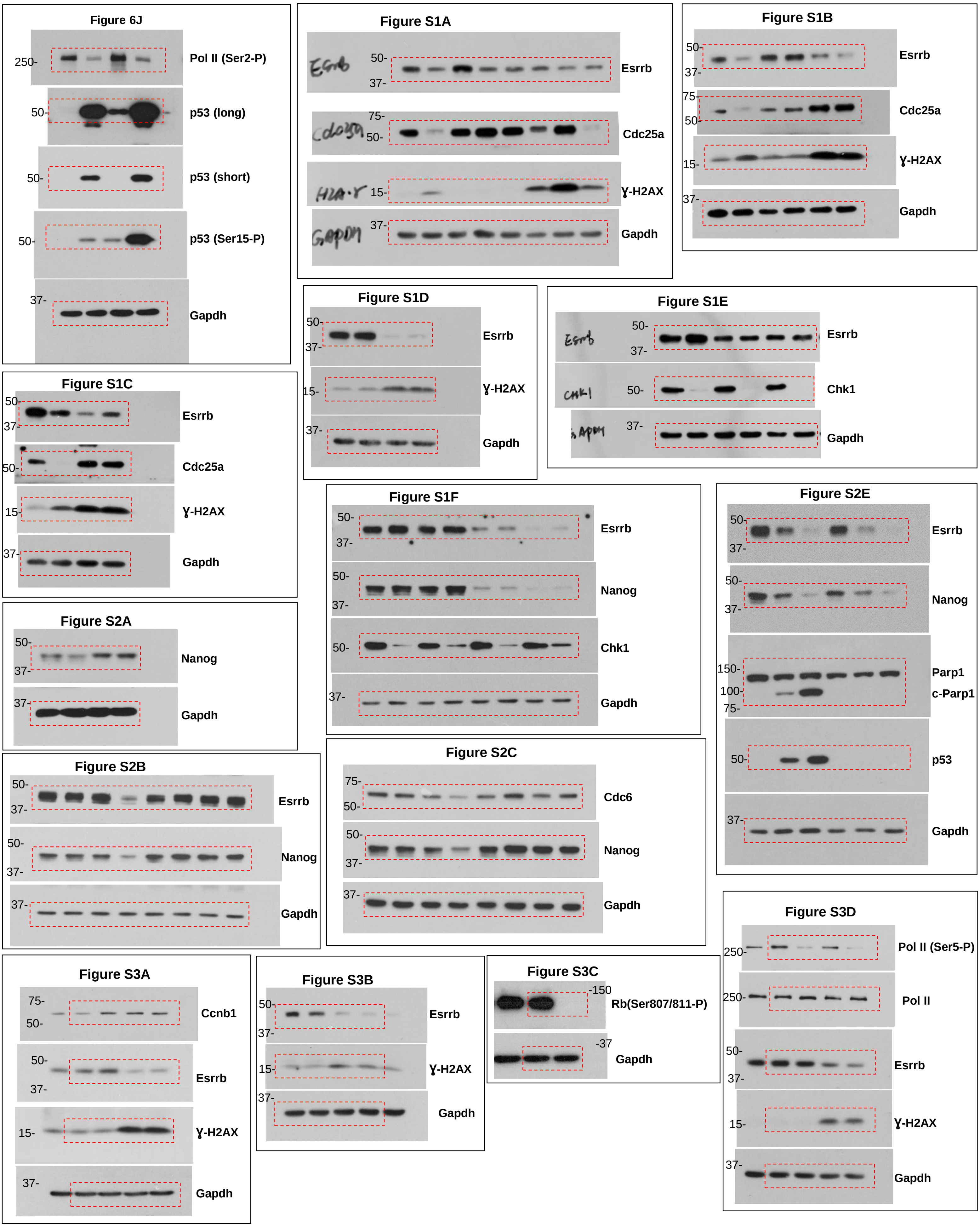
Figure 6J
250-
Pol II (Ser2-P)
50-
p53 (long)
50-
p53 (short)
50-
p53 (Ser15-P)
37-
Gapdh
Figure S1A
50-
37-
Esrrb
75-
50-
Cdc25a
15-
Ɣ-H2AX
37-
Gapdh
Figure S1B
50-
37-
Esrrb
75-
50-
Cdc25a
15-
Ɣ-H2AX
37-
Gapdh
Figure S1C
50-
37-
Esrrb
50-
Cdc25a
15-
Ɣ-H2AX
37-
Gapdh
Figure S1D
50-
37-
Esrrb
15-
Ɣ-H2AX
37-
Gapdh
Figure S1E
50-
37-
Esrrb
50-
Chk1
37-
Gapdh
Figure S1F
50-
37-
Esrrb
50-
37-
Nanog
50-
Chk1
37-
Gapdh
Figure S2E
50-
37-
Esrrb
50-
37-
Nanog
150-
100-
75-
Parp1
c-Parp1
50-
p53
37-
Gapdh
Figure S2A
50-
37-
Nanog
37-
Gapdh
Figure S2B
50-
37-
Esrrb
50-
37-
Nanog
37-
Gapdh
Figure S2C
75-
50-
Cdc6
50-
37-
Nanog
37-
Gapdh
Figure S3D
250-
Pol II (Ser5-P)
250-
Pol II
50-
37-
Esrrb
15-
Ɣ-H2AX
37-
Gapdh
Figure S3A
75-
50-
Ccnb1
50-
37-
Esrrb
15-
Ɣ-H2AX
37-
Gapdh
Figure S3B
50-
37-
Esrrb
15-
Ɣ-H2AX
37-
Gapdh
Figure S3C
-150
Rb(Ser807/811-P)
-37
Gapdh

Figure S3E

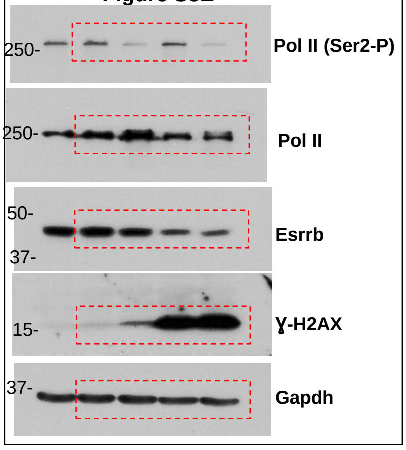

Figure S4

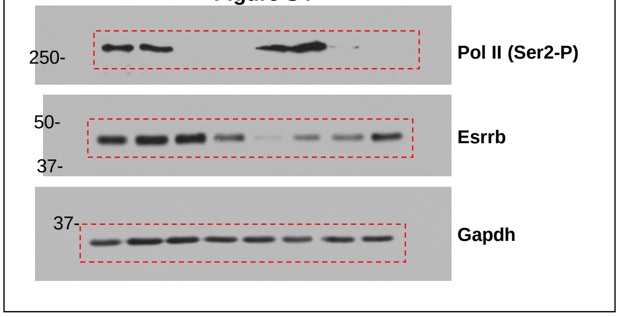

Figure S5A

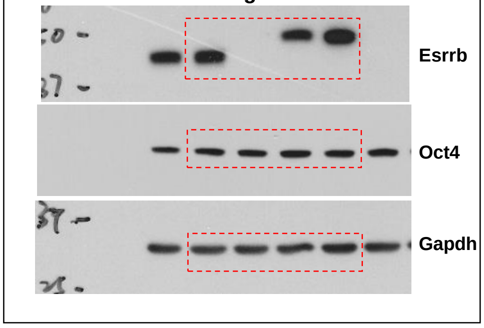

Figure S9B

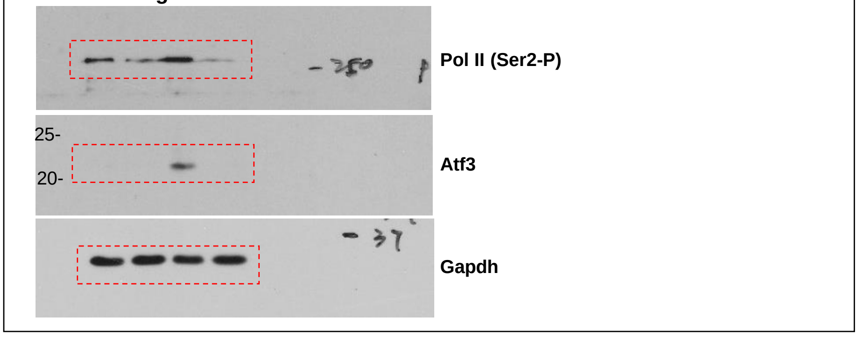

Figure S9D

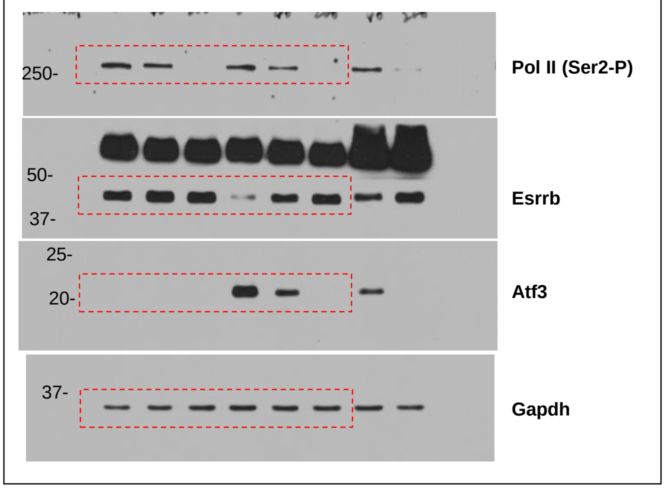

Figure S9E

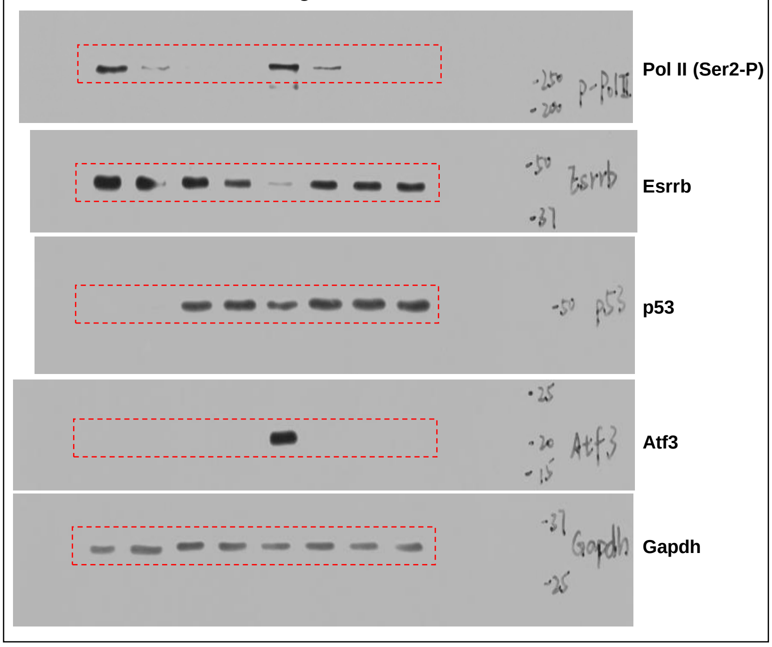

Figure S9F

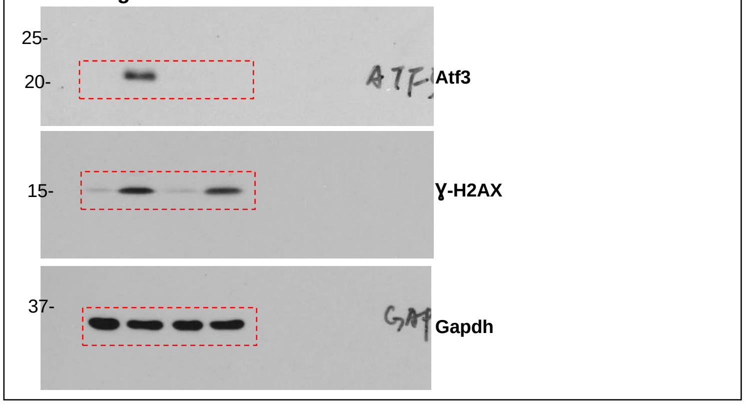

Figure S9J

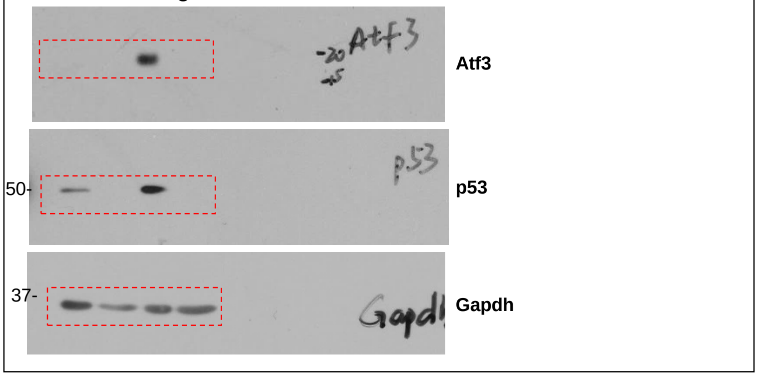

Figure S9H

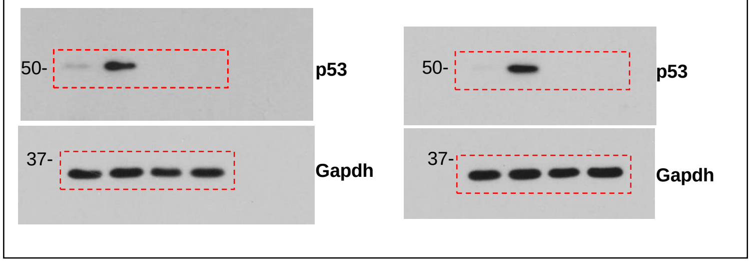

Supplement: Document S1. Figures S1–S12 and Table S1 and Data S1 [file mmc1.pdf]
